# Rapamycin Reduces Amyloid‐β Plaques and Improves Behavioral Performance in a Sex‐Dependent Manner in Mouse Models of Amyloidosis

**DOI:** 10.1002/cns.70807

**Published:** 2026-02-26

**Authors:** Shihui Guo, Weishan Fu, Yating Wang, Qi Liu, Jiaxin Li, Kai Guo, Hongsheng Zhang

**Affiliations:** ^1^ Department of Neurobiology, School of Basic Medical Sciences Chongqing Medical University Chongqing China; ^2^ Key Laboratory of Major Brain Disease and Aging Research (Ministry of Education) The First Affiliated Hospital of Chongqing Medical University Chongqing China; ^3^ Department of Psychiatry The First Affiliated Hospital of Chongqing Medical University Chongqing China; ^4^ Department of Neurology The First Affiliated Hospital of Chongqing Medical University Chongqing China

**Keywords:** Alzheimer's disease, lipid droplet, lysosome, microglia, rapamycin

## Abstract

**Background:**

Alzheimer's disease (AD), the most common form of dementia, lacks effective disease‐modifying treatments. Rapamycin, an mTOR inhibitor with immunomodulatory properties, may mitigate AD pathology by restoring microglial functions.

**Methods:**

Rapamycin was orally administered to 2‐month‐old 5xFAD and hAPP^NL^.

**Results:**

Rapamycin treatment reduced the cerebral Aβ plaque burden, alleviated dystrophic neurites, suppressed glial hyperactivation, and increased plaque‐associated microglial density in both mouse models, with more pronounced effects in female mice. These pathological improvements were associated with attenuated deficits in hippocampal‐dependent memory tasks (spontaneous alternation in the Y‐maze and contextual fear conditioning tasks). Mechanistically, rapamycin enhances microglial lysosomal degradation, promotes lipid droplet clearance in BV2 cells, and increases Aβ phagocytic clearance in primary microglial cells.

**Conclusions:**

Our findings suggest that rapamycin reduces amyloid pathology and associated behavioral deficits in AD mice, an effect associated with enhanced microglial lysosomal activity and Aβ clearance, highlighting its therapeutic potential in AD treatment.

## Graphical abstract

Rapamycin treatment reduced cerebral Aβ plaque burden, alleviated neuritic dystrophy, suppressed glial hyperactivation, and increased plaque‐associated microglial recruitment in amyloidosis mouse models, resulting in improved memory performance. Mechanistically, rapamycin enhanced microglial lysosomal degradation, facilitated lipid droplet clearance in BV2 cells, and promoted Aβ phagocytosis in primary microglia.

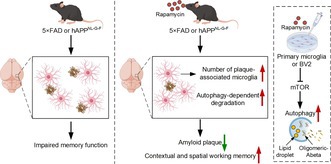

**Abbreviations**
AD: Alzheimer's disease
Aβ: amyloid‐β
CQ: chloroquine
DEGs: differentially expressed genes
FBS: fetal bovine serum
GM‐CSF: granulocyte‐macrophage colony‐stimulating factor
GO: Gene Ontology
IF: immunofluorescence
KEGG: Kyoto Encyclopedia of Genes and Genomes
LAPM1: lysosomal‐associated membrane protein
OA: oleic acid
OFT: Open Field Test
PBST: PBS containing 0.25% Triton X‐100
WB: western blot

## 1 Background

Alzheimer's disease (AD), the leading cause of dementia, is an escalating global health challenge owing to the aging population [1]. Pathologically, AD is characterized by extracellular amyloid‐β (Aβ) plaques and intracellular neurofibrillary tangles composed of hyperphosphorylated tau, which together disrupt synaptic function, impair neuronal communication, and provoke chronic neuroinflammation, culminating in progressive cognitive decline [2, 3, 4].

Current treatments, such as acetylcholinesterase inhibitors (e.g., donepezil) and N‐methyl‐D‐aspartate (NMDA) receptor antagonists (e.g., memantine), provide only modest symptomatic relief [5]. Recently approved anti‐Aβ antibodies, including lecanemab and donanemab, can reduce plaque burden but offer limited benefits in the late stages of the disease [6]. These limitations underscore the urgent need for disease‐modifying therapies that target early pathological mechanisms.

Rapamycin, an mTOR inhibitor initially developed as an immunosuppressant, has attracted attention for its ability to extend lifespan and ameliorate age‐related disorders by inducing autophagy, a lysosomal degradation pathway that clears damaged organelles and aggregated proteins [7, 8]. In AD models, rapamycin reduces Aβ and tau pathologies, restores synaptic integrity, and improves cognition [9, 10, 11, 12, 13]. However, the mechanisms underlying these effects, particularly their effects on microglial function, remain to be elucidated.

Microglial cells are resident immune cells in the brain that play a central role in Aβ clearance. In AD, sustained inflammatory activation drives microglial dysfunction, reduces phagocytic capacity, and exacerbates neurodegeneration [14, 15]. Restoring microglial homeostasis by modulating lipid metabolism, phagocytosis, and inflammatory signaling has emerged as a promising therapeutic strategy [16, 17, 18]. Given the immunomodulatory and autophagy‐enhancing properties of rapamycin, we hypothesized that its therapeutic benefits in AD may involve the restoration of microglial function.

In this study, we investigated the effects of early rapamycin treatment in two complementary AD mouse models (5xFAD and hAPP^NL‐G‐F^). We assessed the impact of this compound on Aβ deposition, neuroinflammation, and cognition and examined its direct effects on microglia using primary cultures and cell lines. By integrating behavioral, histopathological, and molecular analyses, we provide new evidence that rapamycin mitigates AD pathology, at least in part, via microglial modulation.

## 2 Materials and Methods

### 2.1 Study Design

This study employed a preclinical interventional design using two distinct AD mouse models: 5xFAD and hAPP^NL‐G‐F^ mice. Both male and female mice were used in all experiments to assess potential sex differences. At 2 months of age, during the early phase of Aβ pathology, the animals were administered rapamycin or vehicle‐supplemented chow daily for 90 days. Comprehensive behavioral assessments (Open Field Test, Y‐maze, and Contextual Fear Conditioning) were conducted at approximately 5 months of age. Following behavioral testing, the mice were euthanized, and their brain tissues were collected for analysis. The analytical methodologies encompassed (1) quantitative evaluation of AD pathology through x34‐stained plaque analysis and dystrophic neurite quantification; (2) neuroinflammatory assessment via examination of CD68/IBA1‐positive microglial activation and GFAP‐positive astrogliosis; and (3) mechanistic investigations involving cortical tissue RNA sequencing, microglial plaque colocalization studies, and detection of autophagic substrate P62. In vitro cellular experiments included: (1) treatment of primary microglia with fluorophore‐conjugated oligomeric Aβ (pHrodo‐ or FAM‐labeled Aβ), either alone or in combination with rapamycin, to evaluate microglial phagocytic and degradative capacity, and (2) administration of rapamycin or autophagy inhibitors to oleic acid‐pretreated BV2 microglial cell lines to analyze lipid droplet clearance mechanisms.

### 2.2 Animals

This study used heterozygous 5xFAD murine models with a C57BL/6‐SJL hybrid genetic background obtained from Jackson Laboratory (MMRRC stock #34840‐JAX) [19]. These models were engineered to co‐express pathogenic human amyloid precursor protein variants (K670N/M671L Swedish mutation, I716V Florida substitution, and V717I London alteration) and presenilin‐1 mutations (M146L/L286V), all of which are regulated by the neuron‐specific murine Thy‐1 promoter. Additionally, hAPP^NL‐G‐F^ knock‐in mice were obtained from C57BL/6Smoc‐Apptm3(hAPP^NL‐G‐F^)/Smoc (#NM‐HU‐2000088) [20]. These mice express Swedish “NL,” Iberian “F,” and Arctic “G” mutations in the APP gene under an endogenous promoter on a C57BL/6J background.

All mice were housed in a barrier facility at the Chongqing Medical University Animal House under controlled conditions: 12 h light–dark cycle, 40%–70% humidity, and 20°C–26°C. Two‐month‐old 5xFAD or hAPP^NL‐G‐F^ mice were randomly assigned to two groups: vehicle control group, standard diet with microencapsulation carrier; rapamycin‐treated group, custom diet containing 143 ppm rapamycin (14 ppm active compound; Changzhou Haijiakanghui Biotechnology Co. Ltd.), delivering 2.33 mg/kg/day based on a 30 g body weight and 5 g daily intake. The mice were given ad libitum access to food and water for 3 months. All mice that underwent behavioral testing were euthanized at the experimental endpoint (approximately 4.7 months of age), and their brain tissues were collected for subsequent immunofluorescence and biochemical analyses. All animal procedures used in this study were conducted in accordance with the National Institutes of Health Guidelines for the Care and Use of Laboratory Animals and were approved by the Institutional Animal Care and Use Committee of Chongqing Medical University (IACUC‐CQMU‐2024‐0931).

### 2.3 Open Field Test (OFT)

The OFT was conducted following established protocols with minor modifications [21]. Briefly, the mice were acclimated to the testing room for 2 h before the experiment. Each mouse was placed in a corner of the peripheral zone within a square arena (40 × 40 cm with 30‐cm‐high walls) and allowed to explore freely for 10 min. The central zone was defined as a 20 × 20 cm area, and the peripheral zone comprised the transition area from the edge to the central region. An overhead camera recorded the session. The total distance traveled was used to quantify locomotor activity, and the time spent in the center zone was used to evaluate anxiety‐like behavior.

### 2.4 Y‐Maze

The spontaneous alternation test in a Y‐maze was used to evaluate short‐term spatial learning and memory, following established protocols with minor methodological modifications to optimize behavioral assessment [22]. Before behavioral testing, the mice were acclimated to the experimental room for 2 h. The mice were allowed to freely explore the Y‐maze apparatus with three equally angled arms (30 cm × 8 cm × 15 cm) for 8 min each. Alternation was defined as consecutive entries into three distinct arms, and the alternation percentage was calculated as follows: [number of alternations/(total arm entries −2)] × 100.

### 2.5 Fear Conditioning

Fear conditioning was conducted according to the established protocols [23]. On the training day, the mice were placed in conditioning chambers for a 2‐min acclimation period, followed by three tone–shock pairings. Each pairing consisted of a 30‐s white noise (75 dB, 2 kHz; conditioned stimulus) that co‐terminated with a 2‐s foot shock (0.5 mA; unconditioned stimulus) and a 1.5‐min interstimulus interval between pairings. After the final pairing, the mice were kept in the chamber for an additional 1.5 min before being returned to their home cages. Twenty‐four hours later, contextual fear memory was assessed by placing the mice back into the same chamber (no tone/shock) for 5 min, during which freezing behavior, defined as complete immobility except for respiration, was recorded. Four hours after the context test, cued fear memory was evaluated in a novel chamber using distinct contextual cues (vanilla scent and fluorescent lights). This test lasted 6 min; after a 3‐min adaptation period, the conditioning tone was presented for the final 3 min, and freezing behavior was recorded. All sessions were video recorded using overhead cameras, and the freezing behavior was automatically quantified using TopScan software (Clever Sys Inc., USA). The chambers were thoroughly cleaned and sanitized before the trials.

### 2.6 X34 Staining for Amyloid Plaques and Quantification

X34 staining and quantification were performed as previously described [24] by an investigator blinded to the genotype. Coronal sections (40 μm) spanning the hippocampus (−0.95 to −3.40 mm from Bregma) were collected at 240 μm intervals. Eight to ten anatomically matched sections per animal were analyzed, with section matching validated using anatomical landmarks, including the lateral ventricle and corpus callosum morphology.

The sections were washed thrice with PBS (5 min each), permeabilized with 0.3% Triton X‐100 in PBS for 30 min, and incubated in a solution of 40% ethanol (EtOH) and 60% PBS containing 1 μM X34 (Merck, #SML1954) for 20 min. After washing in X34 buffer (40% EtOH in PBS) and PBS, the sections were mounted and sealed using Fluormount‐G (Southern Biotech, 0100‐01), and stored in the dark at 4°C until imaging.

Plaque quantification was performed as previously described [25], with minor modifications. Images were acquired using a spinning‐disk confocal microscope and processed using the Olympus CellSens Viewer (Olympus Corporation). Images were converted to 8‐bit grayscale using ImageJ software to facilitate analysis. A threshold was applied to enhance plaque visualization while minimizing background noise, and each object was manually reviewed to confirm plaque identity. 8–10 brain sections were analyzed for each mouse. Quantification was performed separately for the cortex and hippocampus. Regions of interest (ROI) were manually outlined in ImageJ, and only plaques larger than 5.5 μm^2^ were included. The total areas of the brain sections (ROIs) were measured to ensure accurate scaling and enable comparisons across samples.

### 2.7 Immunofluorescence

Coronal brain sections (40‐μm‐thick) from one hemisphere were washed three times with PBS, then blocked and permeabilized with PBST and 5% bovine serum albumin (BSA) for 1 h at room temperature. Primary antibodies were diluted in blocking buffer according to the manufacturer's instructions and incubated with the sections overnight at 4°C. The following day, after removing the primary antibodies and washing three times with PBS, fluorescence‐conjugated secondary antibodies were applied and incubated for 1 h at room temperature in the dark. After three additional PBS washes, the sections were mounted using an antifade mounting medium (Solarbio, S2100) and imaged using a confocal laser‐scanning microscope (Olympus SpinSR). Quantitative analysis was performed using ImageJ software (NIH).

For the statistical analysis of immunofluorescence, 5–6 mice per group (encompassing both sexes) were used, with brain sections of comparable hippocampal size selected for staining and quantification. Briefly, multiple key metrics were assessed to evaluate the neuroinflammation and autophagy status. To assess autophagic flux in plaque‐associated microglia, the mean fluorescence intensity of p62 (an autophagic marker) and phosphorylated S6 (pS6, a downstream target of mTOR) was measured directly within IBA1^+^ cells in direct contact with Aβ plaques. Furthermore, the proportion of the CD68^+^ area within the IBA1^+^ microglia was quantified across the entire brain section as an indicator of phagocytic activation in the brain. Additionally, the percentages of LAMP1^+^ structures and GFAP^+^ astrogliosis relative to the total brain area were calculated. Finally, PU.1^+^ nuclei were counted in three representative fields of view encompassing both the cortex and hippocampus in each section. To specifically examine the peri‐plaque immune response, the number of PU.1^+^ cells surrounding the Aβ plaques was quantified, with 10 randomly selected plaques of similar size analyzed in each section. The antibodies used for immunofluorescence are listed in Table 1.

**TABLE 1:** Antibodies used for immunofluorescence and Western blot.

Antibodies	Source	Identifier
Phospho‐S6 (Ser235)	Proteintech	Cat#67898‐1‐Ig; RRID: AB_2918654
CD68	BIO‐RAD	Cat#MCA1957; RRID: AB_3100585
IBA1	Oasis	Cat#OB‐PGP049‐01; RRID: AB_2934253
IBA1	Novus	Cat#NB100‐1028; RRID: AB_3148646
LAMP1	DSHB	Cat#1D4B; RRID: AB_528127
GFAP	Abclonal	Cat#A19058; RRID: AB_2862551
PU.1	Cell Signaling Technology	Cat#2258S; RRID: AB_2186909
mTOR	Cell Signaling Technology	Cat#2983; RRID: AB_2105622
Phospho‐mTOR (Ser2448)	Cell Signaling Technology	Cat#5536; RRID: AB_10691552
P62	Proteintech	Cat#18420–1‐AP; RRID: AB_10694431
LC3	Proteintech	Cat#14600‐1‐AP; RRID: AB_2137737
β‐Actin	Proteintech	Cat#66009‐1‐Ig; RRID: AB_2687938
GAPDH	Proteintech	Cat#60004‐1‐Ig; RRID: AB_2107436
Tubulin‐α	Proteintech	Cat#80762‐1‐RR; RRID: AB_2918911
HRP‐conjugated Anti‐Rabbit IgG (H + L)	Proteintech	Cat#SA00001‐2; RRID: AB_2722564
HRP‐conjugated Anti‐Mouse IgG (H + L)	Proteintech	Cat#SA00001‐1; RRID: AB_2722565
Anti‐Rabbit IgG (H + L); Alexa Fluor 488	Invitrogen	Cat#A‐21206; RRID: AB_2535792
Anti‐Rabbit IgG (H + L); Alexa Fluor 568	Invitrogen	Cat#A10042; RRID: AB_2534017
Anti‐Rabbit IgG (H + L); Alexa Fluor 647	Invitrogen	Cat#A‐31573; RRID: AB_2536183
Anti‐Goat IgG (H + L); Alexa Fluor 647	Invitrogen	Cat#A‐21447; RRID: AB_2535864
Anti‐Rat IgG (H + L); Alexa Fluor 488	Invitrogen	Cat#A48269; RRID: AB_2893137
Anti‐Rat IgG (H + L); Alexa Fluor 647	Invitrogen	Cat#A21247; RRID: AB_141778
Anti‐Guinea pig IgG (H + L); Alexa Fluor 555	Invitrogen	Cat#A21435; RRID: AB_2535856

### 2.8 Primary Microglial and BV2 Cell Cultures

Primary microglia were isolated from postnatal day 1–3 WT mouse pups according to an established methodology [26]. Following meningeal removal in ice‐cold HBSS, the dissected cortical and hippocampal tissues were enzymatically digested with 0.25% trypsin–EDTA at 37°C for 20 min. The resulting cell suspension was filtered through a 70‐μm nylon mesh and centrifuged at 800 *g* for 5 min before resuspension in a microglial culture medium consisting of DMEM supplemented with 10% fetal bovine serum, 1% penicillin–streptomycin, and 50 ng/mL recombinant murine granulocyte‐macrophage colony‐stimulating factor (GM‐CSF; PeproTech, AF‐300‐03). Cells were plated onto poly L‐lysine‐coated T75 flasks, and two‐thirds of the medium was replaced every 3 days. After 9–10 days of culture, the microglia were detached by orbital shaking at 200 rpm for 30 min. The cells were collected by centrifugation, resuspended in fresh complete medium, and plated in 24‐well plates for subsequent experiments.

BV2 cells were cultured in DMEM containing 10% FBS and 1% penicillin/streptomycin (100 U/mL penicillin and 100 μg/mL streptomycin) at 37°C/5% CO_2_. Upon reaching 80% confluence, the cells were trypsinized and passaged into 6‐well or 24‐well plates for further experimentation. Rapamycin (MCE, HY‐10219), oleic acid (Sigma, O1008), and chloroquine (MCE, HY‐17589A) were administered to the experimental groups.

### 2.9 Protein Extraction and Western Blot Assay

BV2 cells and mouse cortical tissues were lysed on ice for 10 min using RIPA buffer (Beyotime, P0013) containing complete protease and phosphatase inhibitors. Cell lysates were sonicated (40 W, 1 min; BV2 cells) or homogenized (40 Hz, 1 min; tissues) and centrifuged at 8000 *g* for 10 min at 4°C. Protein concentrations were determined using a BCA assay kit (Thermo Fisher Scientific, 23225). Equal amounts of proteins were separated on 8% or 12% SDS‐PAGE gels, transferred to PVDF membranes, blocked with 5% nonfat milk/BSA, and incubated with primary antibodies overnight at 4°C. After incubation with horseradish peroxidase‐conjugated secondary antibodies (1:10,000 in 5% bovine serum albumin/TBST), bands were visualized using a Super‐sensitive ECL chemiluminescent substrate (Biosharp, BL520A) and quantified relative to GAPDH/β‐actin using ImageJ (NIH). The antibodies used for western blotting are listed in Table 1.

### 2.10 RNA Sequencing Analysis

Total RNA was isolated from mouse cortical tissues (5xFAD Male and Female) using TRIzol reagent (Thermo Fisher, 15596026) and purified using the Direct‐zol RNA MiniPrep Kit (Zymo Research, R2052). RNA sequencing (RNA‐seq) was performed by Novogene (Beijing, China). The mRNA was enriched using oligo (dT)‐attached magnetic beads, fragmented, and reverse‐transcribed to cDNA. Libraries were prepared by end repair, adenylation, adapter ligation, size selection (150–200 bp), and polymerase chain reaction (PCR) amplification. Sequencing was performed on an Illumina HiSeq 2500 platform (150 bp paired‐end). Raw sequencing reads were assessed for quality using FastQC (v0.11.9). Adapter sequences and low‐quality bases were removed using Trimmomatic (v0.39), with the first 10 bp of each read trimmed to eliminate potential bias. The cleaned reads were aligned to the mouse reference genome (GRCm38/mm10) using HISAT2 (v2.2.1) with default parameters. Gene‐level read counts were generated using featureCounts (v2.0.3) with Ensembl gene annotation. Differential gene expression analysis was performed using DESeq2 (v1.38.3) to identify significantly altered genes, and differentially expressed genes (DEGs) were selected based on an adjusted *p*‐value (*p*adj) < 0.05 and absolute fold change ≥ 1.5. When subjected to model‐based clustering (Mfuzz v2.66.0), genes were grouped based on their expression patterns using the parameters *c* = 5 and *m* = 1.19. Functional enrichment (GO, KEGG) was analyzed using richR (https://github.com/hurlab/richR), and significant pathways were selected based on a *p*‐value < 0.05.

### 2.11 Lipid Droplet Staining

BV2 cells were fixed with 4% paraformaldehyde (20 min, RT), permeabilized with BODIPY buffer (PBS/0.1% saponin/1% BSA; 30 min, RT), blocked with 10% donkey serum in BODIPY buffer (1 h, RT), and incubated with 0.1 μg/mL BODIPY493/503 (Invitrogen, D2191; 45 min, 37°C). After washing with PBS, the nuclei were counterstained with DAPI (1 μg/mL). Images were acquired using a confocal microscope (Olympus Spin SR).

### 2.12 Flow Cytometry

Flow cytometry was used to assess the lipid droplet content in BV2 cells [27]. The cells were trypsinized, pelleted, stained with 0.03 μg/mL BODIPY493/503 (15 min, RT), washed with PBS, and analyzed using a flow cytometer (SONY SA3800; FITC channel). Data were processed using FlowJo (BD Life Sciences).

### 2.13 Primary Microglia Phagocytosis Assay

To evaluate the effect of rapamycin on microglial phagocytic activity, primary microglial cells were seeded at a density of 2 × 10^4^ cells/well in 24‐well plates. After preincubation for 24 h with either 200 nM Aβ_1‐42_ alone or a combination of 100 nM rapamycin and 200 nM Aβ_1‐42_, the medium was replaced with a serum‐free medium containing 0.5 mg/mL pHrodo Red Zymosan BioParticles (Invitrogen, Cat# P35364). The cells were monitored in real time using an IncuCyte Live Cell Imaging System (Sartorius). The phagocytic rate was quantified by calculating the percentage of red fluorescent cells at 30, 60, and 120 min.

### 2.14 Primary Microglia Aβ Degradation Assay

To assess the effect of rapamycin on the Aβ‐degrading capacity of primary microglia, the cells were plated in 24‐well plates containing sterile coverslips. Two experimental groups were established: the FAM‐Aβ (ANASpec, AS‐23525‐05) group (treated with 200 nM FAM‐Aβ) and the FAM‐Aβ + Rapamycin group (pretreated with 100 nM rapamycin for 24 h before the addition of 200 nM FAM‐Aβ). At 30, 60, and 120 min after FAM‐Aβ administration, the culture medium was removed, and the cells were washed three times with PBS to eliminate residual extracellular FAM‐Aβ. The cells were fixed with 4% paraformaldehyde (PFA) and counterstained with DAPI to visualize the nuclei. Images were acquired using a confocal microscope (Olympus Spin SR).

### 2.15 Statistical Analysis

Data represent ≥ 3 biologically independent replicates (mean ± S.E.M.). Outliers were removed using the ROUT method (*Q* = 1%; GraphPad Prism 9.0). The choice of statistical test was based on the data distribution and the homogeneity of the variances. For comparisons between two independent groups, an unpaired two‐tailed Student's *t*‐test was used when the assumptions of normality and equal variances were met; Welch's *t*‐test was applied when variances were unequal despite normality; and the Mann–Whitney *U* test was used when the data violated normality assumptions. Multiple unpaired *t*‐tests were performed with Welch's correction for multiple comparisons between the two groups across several variables, and significance was determined based on false discovery rate (FDR)‐adjusted *q*‐values using the two‐stage step‐up method of Benjamini, Krieger, and Yekutieli (*Q* = 5%). One‐way ANOVA was used for comparisons between more than two groups. Effect sizes were calculated as Cohen's *d* or *η* ^2^ and are reported in the figure legends. No statistical analyses were performed to determine the sample size. The number of biological replicates is specified in the figure legend. Statistical significance was defined as *p* < 0.05 for single comparisons and *q* < 0.05 for multiple comparisons. The significance levels are denoted as follows: * *p* < 0.05, ** *p* < 0.01, *** *p* < 0.001, and **** *p* < 0.0001. Details of the biological replicates are provided in the figure legends.

## 3 Results

### 3.1 Dietary Rapamycin Reduces mTOR Activity and Downstream Signaling in AD Mice

**FIGURE 1:**
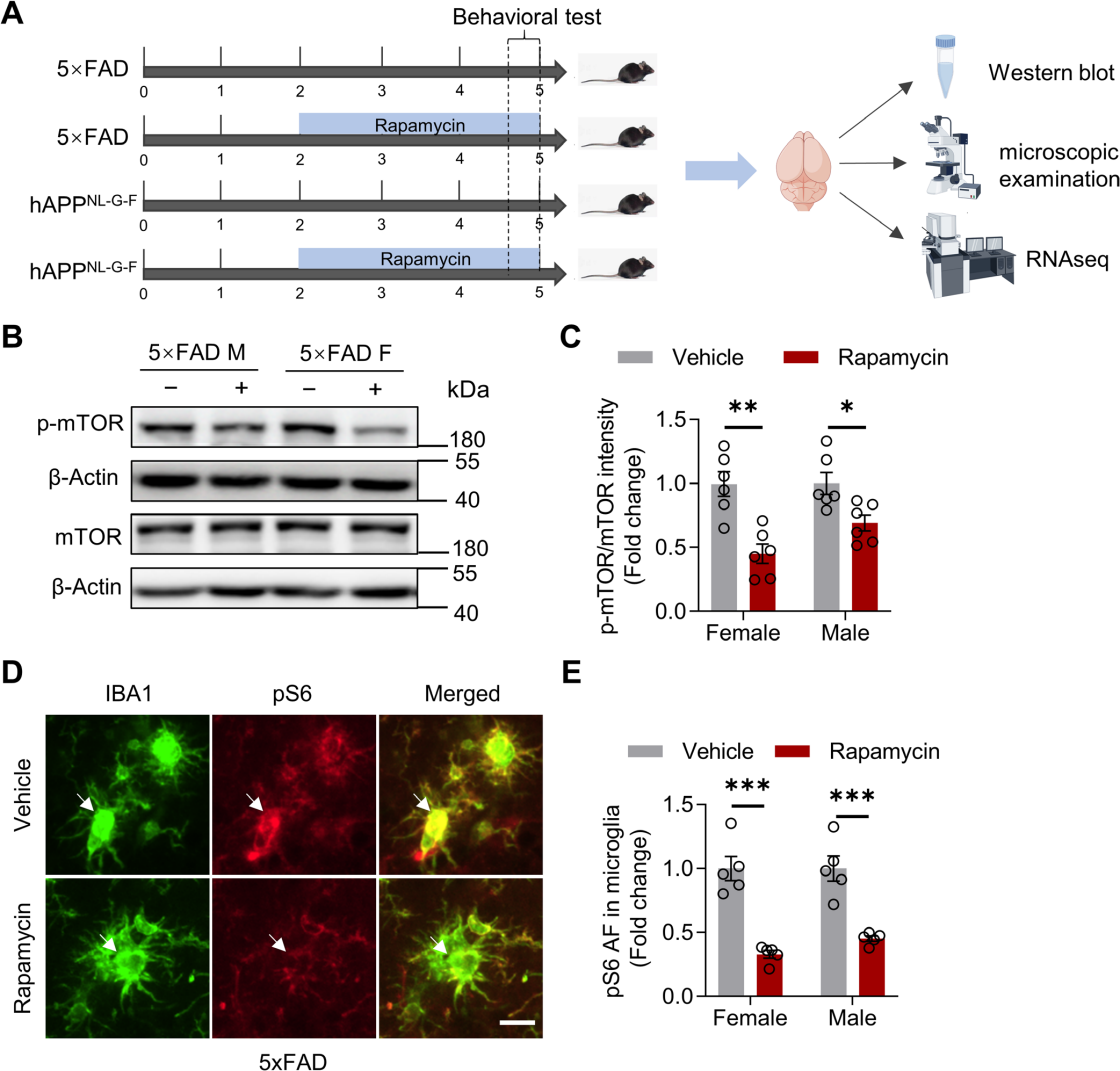
Reduced mTOR activity and downstream signaling in rapamycin‐treated 5xFAD mice. (A) Experimental design for intermittent dietary rapamycin administration (starting at 2 months of age) in AD‐model mice. (B, C) Immunoblot and quantification showing total mTOR and phosphorylated mTOR (p‐mTOR) levels in the cortex of vehicle‐ or rapamycin‐treated 5xFAD mice. *n* = 6 mice per group. Female: *T*_(10)_ = 4.472, *p* = 0.0012; male: *T*_(10)_ = 2.922, *p* = 0.0153, unpaired *t*‐test. (D, E) Confocal images and quantification showing phosphorylated S6 ribosomal protein (pS6, red) in Iba1‐positive microglia (green) proximal to Aβ plaques in the brains of vehicle‐ or rapamycin‐treated 5xFAD mice. Arrows indicate pS6‐positive microglia surrounding the plaques. Scale bar, 10 μm. *n* = 5 mice per group. Female: *T*_(8)_ = 6.704, *p* = 0.0002; male: *T*_(8)_ = 5.426, *p* = 0.0006, unpaired *t*‐test. Data are mean ± SEM. *p < 0.05, **p < 0.01, ***p < 0.001.

To investigate the effect of rapamycin on AD pathogenesis, we administered dietary rapamycin to two AD mouse models (5xFAD and hAPP^NL‐G‐F^) starting at 2 months of age. Behavioral assessments were initiated when the mice reached 4.7 months of age. Brain tissues were collected at 5 months for analysis (western blotting, immunofluorescence, and X34 staining) and for RNA sequencing (Figure 1A). Western blot analysis demonstrated that dietary rapamycin did not alter total mTOR protein levels in the brains of either female or male 5xFAD or hAPP^NL‐G‐F^ mice (Figure 1B,C; Figure S1A,B). However, it significantly reduced the levels of phosphorylated mTOR (p‐mTOR) (Figure 1B,C; Figure S1A,B). Furthermore, immunofluorescence analysis revealed that rapamycin treatment markedly decreased pS6 levels, a downstream target of mTOR, specifically in microglia in both AD models (Figure 1D,E, and Figure S1C,D). Collectively, these results indicate that dietary rapamycin effectively suppresses mTOR activity and its downstream signaling pathways in the brains of two distinct AD mouse models.

### 3.2 Rapamycin Ameliorates Aβ Pathology in AD Mice

**FIGURE 2:**
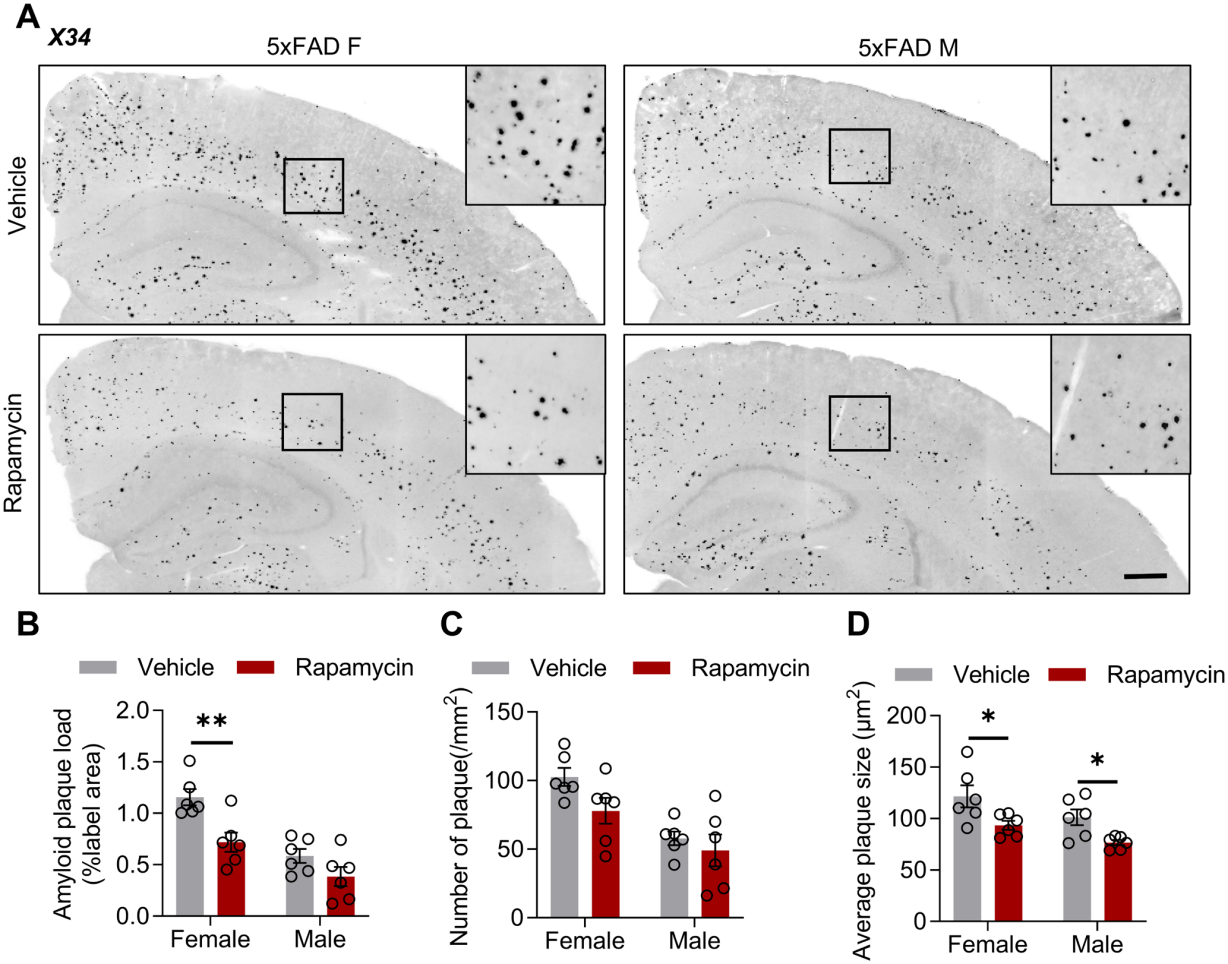
Ameliorated Aβ pathology in rapamycin‐treated 5xFAD mice. (A) Confocal images showing the Aβ plaque (X34) burden in the brains of vehicle‐ or rapamycin‐treated female and male 5xFAD mice. Scale bar, 200 μm. (B–D) Quantification of Aβ plaque load (B), density (C), and average size (D) in vehicle‐ or rapamycin‐treated female and male 5xFAD mice. *n* = 6 mice per group. Amyloid plaque load, female: *T*_(10)_ = 3.549, *p* = 0.0053, male: *T*_(10)_ = 1.734, *p* = 0.1137; number of plaques, female: *T*_(10)_ = 2.134, *p* = 0.0586, male: *T*_(10)_ = 0.7001, *p* = 0.4998; average plaque size, female: *T*_(10)_ = 2.418, *p* = 0.0362, male: *T*_(10)_ = 3.035, *p* = 0.0126; unpaired *t*‐test. Data are mean ± SEM. *p < 0.05, **p < 0.01.

To determine whether rapamycin‐mediated suppression of mTOR activity affects Aβ pathology in AD mice, we performed X34 staining of brain sections from 5xFAD and hAPP^NL‐G‐F^ mice. Analysis revealed that in female 5xFAD mice, rapamycin treatment significantly reduced both Aβ plaque load and plaque size, with plaque numbers showing a decreasing trend (Figure 2A–D). In contrast, male 5xFAD mice exhibited a pronounced reduction in plaque size without alterations in plaque load or number. This sex‐specific disparity may originate from the differential metabolic responses to rapamycin [28]. Parallel experiments in hAPP^NL‐G‐F^ mice demonstrated that rapamycin significantly decreased plaque load and size in both male and female animals, whereas the plaque number remained unchanged (Figure S2A–D). These results demonstrate that rapamycin ameliorates key aspects of Aβ pathology in both AD mouse models.

### 3.3 Rapamycin Mitigates Cognitive Deficits in AD Mice

**FIGURE 3:**
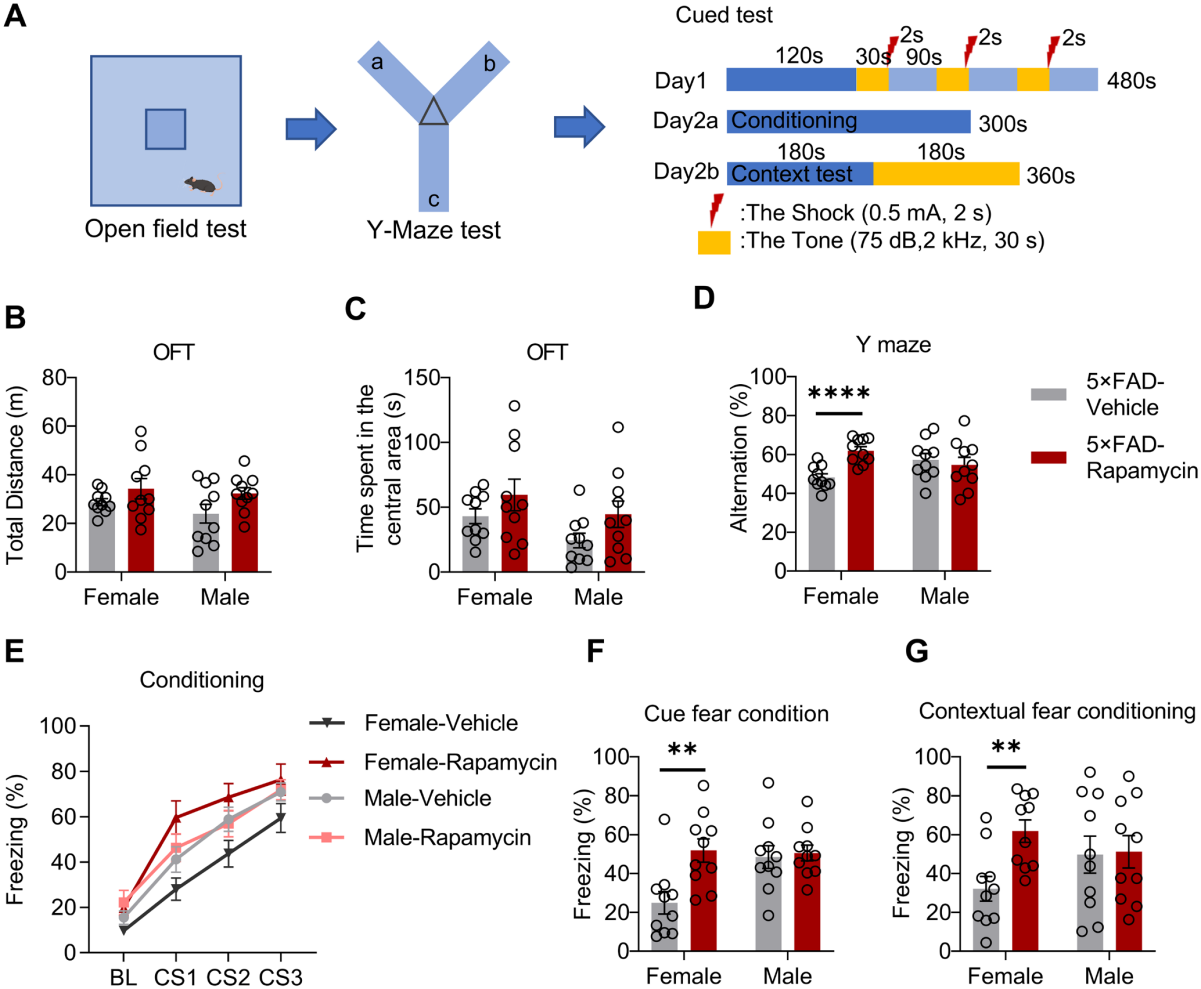
Mitigated cognitive impairment in rapamycin‐treated 5xFAD mice. (A) Schematic diagram and timeline of the behavioral testing. (B, C) Total distance traveled (B) and time spent in the center zone (C) during the open field test in vehicle‐ or rapamycin‐treated 5xFAD mice. Total distance female: *T*_(18)_ = 1.1264, *p* = 0.2225; Total distance male: *T*_(18)_ = 1.861, *p* = 0.0791; time spent in center female: *T*_(18)_ = 1.227, *p* = 0.2357; time spent in center male: *T*_(18)_ = 1.727, *p* = 0.1013; unpaired *t*‐test. (D) Spontaneous alternation percentage during the spontaneous alternation test in a Y‐maze. Female: *T*_(18)_ = 5.037, *p* < 0.0001; male: *T*_(18)_ = 0.5257, *p* = 0.6055, unpaired *t*‐test. (E–G) Freezing percentage during fear conditioning training (E), cued fear conditioning (F), and contextual fear conditioning (G). Cued fear conditioning: Female: *T*_(18)_ = 3.240, *p* = 0.0045; male: *T*_(18)_ = 0.2874, *p* = 0.7771; contextual fear conditioning: Female: *T*_(18)_ = 3.430, *p* = 0.0030; male: *T*_(18)_ = 0.1152, *p* = 0.9095; unpaired *t*‐test. *n* = 10 mice for vehicle, *n* = 10 mice for rapamycin. Data are mean ± SEM. **p < 0.01, ****p < 0.0001.

To assess the effects of rapamycin on cognitive function, we subjected 4.7‐month‐old male and female 5xFAD and hAPP^NL‐G‐F^ mice to a series of behavioral tests, including the open field test (day 1), Y‐maze (day 2), and contextual fear conditioning (day 3–4) (Figure 3A). Sex‐stratified analysis revealed distinct treatment response patterns. Rapamycin did not alter the total distance traveled or time spent in the center zone in the open field test in any group (Figure 3B,C; Figure S3A,B), indicating no effect on general locomotor activity or anxiety‐like behavior. In the Y‐maze, rapamycin significantly improved spontaneous alternation, a measure of spatial working memory, specifically in female mice in both the 5xFAD and hAPP^NL‐G‐F^ models (Figure 3D, Figure S3C). The fear memory outcomes were model‐ and sex‐dependent. In 5xFAD mice, rapamycin significantly increased freezing during both contextual and cued memory tests; however, this effect was exclusive to females (Figure 3E–G). No significant improvement was observed in male 5xFAD mice in either test. In hAPP^NL‐G‐F^ mice, rapamycin did not significantly alter freezing behavior in either sex during contextual or cue testing (Figure S3E,F). Taken together, these data demonstrate that the cognitive benefits of rapamycin, particularly in hippocampal‐dependent tasks, are predominantly evident in female AD model mice. This behavioral sex difference aligns with the more robust reduction in amyloid pathology observed in female 5xFAD mice, suggesting that the efficacy of rapamycin is influenced by model‐specific interactions with sexually dimorphic disease progression.

### 3.4 Rapamycin Reduces Amyloid Plaque‐Associated Toxicity in AD Mice

**FIGURE 4:**
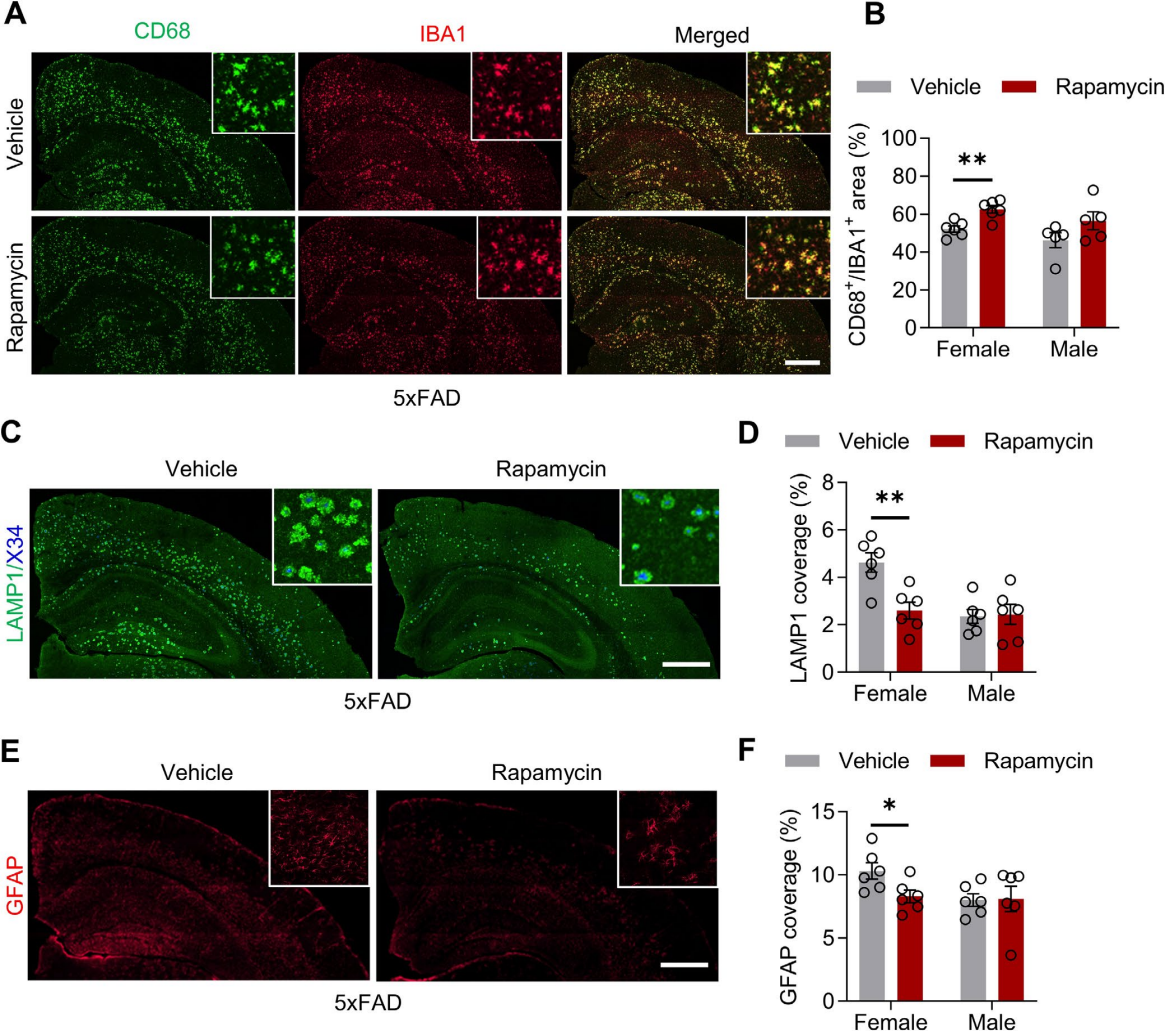
Reduced amyloid plaque‐associated toxicity in rapamycin‐treated 5xFAD mice. (A) Confocal images showing CD68 (green) and Iba1 (red) staining in the brains of vehicle‐ or rapamycin‐treated female and male 5xFAD mice. Scale bar, 500 μm. (B) Quantification of the CD68^+^/Iba1^+^ area ratio. *n* = 5 or 6 mice per group. Female: *T*_(10)_ = 4.063, *p* = 0.0023; male: *T*_(8)_ = 1.657, *p* = 0.1361, unpaired *t*‐test. (C) Confocal images showing LAMP1 (green) staining in the cells. Scale bar, 500 μm. (D) Quantification of the LAMP1^+^ area. *n* = 6 mice per group. Female: *T*_(10)_ = 3.718, *p* = 0.0040; male: *T*_(10)_ = 0.1617, *p* = 0.8748, unpaired *t*‐test. (E) Confocal images showing glial fibrillary acidic protein (GFAP; red) expression. Scale bar, 500 μm. (F) Quantification of the GFAP^+^ area. *n* = 6 mice per group. Female: *T*_(10)_ = 2.481, *p* = 0.0325; male: *T*_(10)_ = 0.08114, *p* = 0.9369, unpaired *t*‐test. Data are mean ± SEM. * *p* < 0.05, **p < 0.01.

Fibrillar Aβ deposition drives plaque‐associated neuritic dystrophy, which is a key contributor to cognitive decline [29]. Given that rapamycin reduces amyloid plaque burden, we investigated whether this was associated with altered microglial phagocytic activity (assessed using the CD68/IBA1 ratio). Immunofluorescence analysis revealed that 3 months of rapamycin treatment significantly increased the CD68^+^/IBA1^+^ ratio in female 5xFAD mice compared to that in control mice, whereas male mice remained unaffected (Figure 4A,B). Female hAPP^NL‐G‐F^ mice exhibited a similar trend, although the increase was not statistically significant. No significant changes were observed in the male mice of either model (Figure S4A,B). These findings indicate that rapamycin enhances microglial phagocytic activity, with a significant effect observed in female 5xFAD mice. Next, we examined amyloid‐associated neuronal toxicity in AD mice by co‐immunostaining for Aβ and lysosomal‐associated membrane protein (LAMP1), a marker of dystrophic neurites [30]. Immunofluorescence analysis demonstrated substantially reduced LAMP1 immunoreactivity in female 5xFAD mice and in both sexes of hAPP^NL‐G‐F^ animals (Figure 4C,D; Figure S4C,D), suggesting that rapamycin treatment reduced the number of dystrophic neurites. Assessment of astrocytic reactivity showed that rapamycin significantly reduced the GFAP + area in female 5xFAD and hAPP^NL‐G‐F^ mice, indicating attenuated neuroinflammation; males showed no change (Figure 4E,F and Figure S4E,F). Together, these findings demonstrate that rapamycin consistently enhances microglial phagocytosis, reduces dystrophic neurites, and suppresses neuroinflammation in AD mouse models, with the most prominent effects in females.

### 3.5 Rapamycin Restores Homeostasis in AD Mice by Regulating Lipid Metabolism and Phagocytosis

**FIGURE 5:**
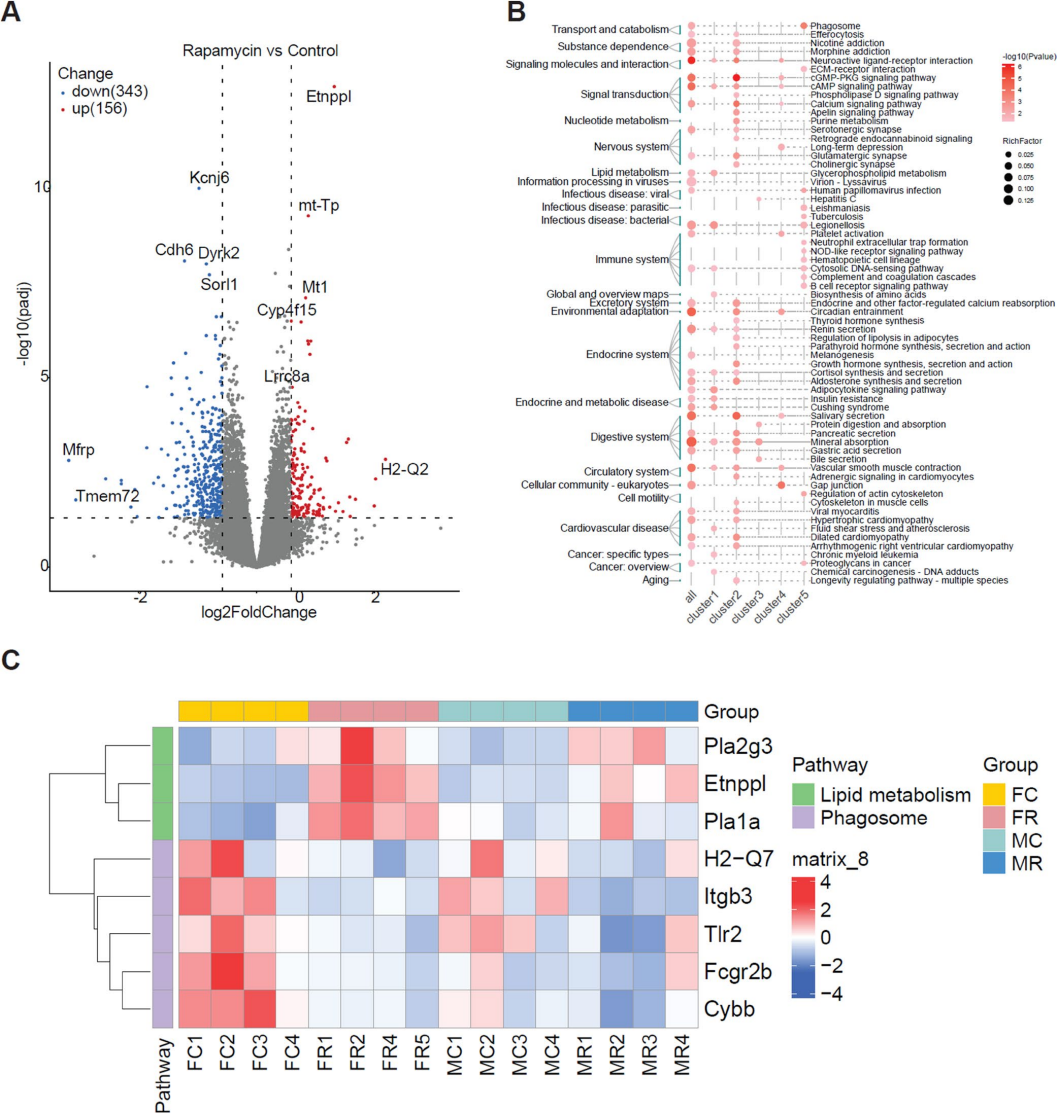
Restored homeostasis in rapamycin‐treated 5xFAD mice via regulation of lipid metabolism and phagocytosis. (A) Volcano plot showing differentially expressed genes (DEGs) in the cortex of vehicle‐ or rapamycin‐treated female and male 5xFAD mice, as determined by RNA‐seq. The *X*‐axis shows log_2_ fold change (FC), and the *Y*‐axis shows −log_10_ of the *p.adj* values. Red and blue dots indicate significantly upregulated or downregulated genes in rapamycin versus vehicle (log_2_ FC > 0.5, *p.adj* value < 0.05). (B) Co‐expression clustering of DEGs using Gaussian mixture modeling. KEGG pathway enrichment analysis revealed significant alterations in the phagosome and lipid metabolism pathways. (C) Hierarchical clustering of lipid metabolism‐related genes (*Pla2g3, Etnppl, Pla1a*) and phagosome‐related genes (*H2‐Q7, Itgb3, Tlr2, Fcgr2b, Cybb*). *n* = 4 mice per group.

To determine how rapamycin ameliorates AD pathology, we performed bulk RNA sequencing of cortical tissues from 5xFAD mice. For this analysis, cortical samples from three female and three male mice per treatment group (vehicle vs. rapamycin) were pooled for sequencing, yielding a total of *n* = 6 biologically independent samples per condition. Initial analysis combining data from both sexes identified 499 differentially expressed genes (DEGs) in response to rapamycin treatment, including 343 downregulated and 156 upregulated transcripts (Figure 5A). When subjected to model‐based clustering, these DEGs were segregated into five distinct co‐expression modules (Figure S5A). These DEGs were segregated into five distinct co‐expression modules (Figure S5A). Functional pathway enrichment analysis of the combined data modules revealed significant associations between phagocytosis and lipid metabolism (Figure 5B).

We separately analyzed the expression profiles to examine the potential sex‐specific transcriptional responses. Hierarchical clustering of key lipid metabolism‐ and phagocytosis‐related genes clearly delineated the samples by both treatment and sex (designated as female control (FC), female rapamycin (FR), male control (MC), and male rapamycin (MR) groups) (Figure 5C). Notably, the magnitude of rapamycin‐induced gene expression changes was more pronounced in female mice than in male mice, consistent with the robust phenotypic improvements observed in females. Specifically, rapamycin promoted the transcription of phospholipase family members (*Pla2g3, Etnppl, and Pla1a*) and downregulated immune‐phagocytic genes (*H2‐Q7, Itgb3, Tlr2, Fcgr2b, and Cybb*), with a stronger effect in the female cohort.

Additionally, RNA‐seq analysis revealed that rapamycin did not significantly affect the expression of Aβ metabolism‐related genes (Bace1, Psen1, App, Psen2, and Bace2) (Figure S5B) or Aβ‐degrading proteases (Mmp2, Mmp9, and Ide) (Figure S5C). Furthermore, using the A8717 antibody, which specifically recognizes the C‐terminus of APP, we measured the levels of APP and its degradation products (CTF‐α/β) in the cerebral cortex of 5xFAD (Figure S6A–C) and hAPP^NL‐G‐F^ (Figure S6D–F) mice. The results demonstrated that rapamycin treatment did not significantly affect the protein levels of APP or CTF‐α/β in either the 5xFAD or hAPP^NL‐G‐F^ mouse models, indicating that its mechanism of action is independent of direct modulation of APP processing or Aβ synthesis/degradation pathways. Taken together, these transcriptional profiles suggest that rapamycin normalizes dysregulated lipid signaling by inhibiting proinflammatory phospholipases and modulating neuroimmune responses by attenuating the phagocytosis pathway, thereby restoring cerebral homeostasis, consistent with its established effects of reducing Aβ deposition and neuroinflammation.

### 3.6 Rapamycin Enhances Lipid Droplet Clearance via Autophagy‐Dependent Degradation

**FIGURE 6:**
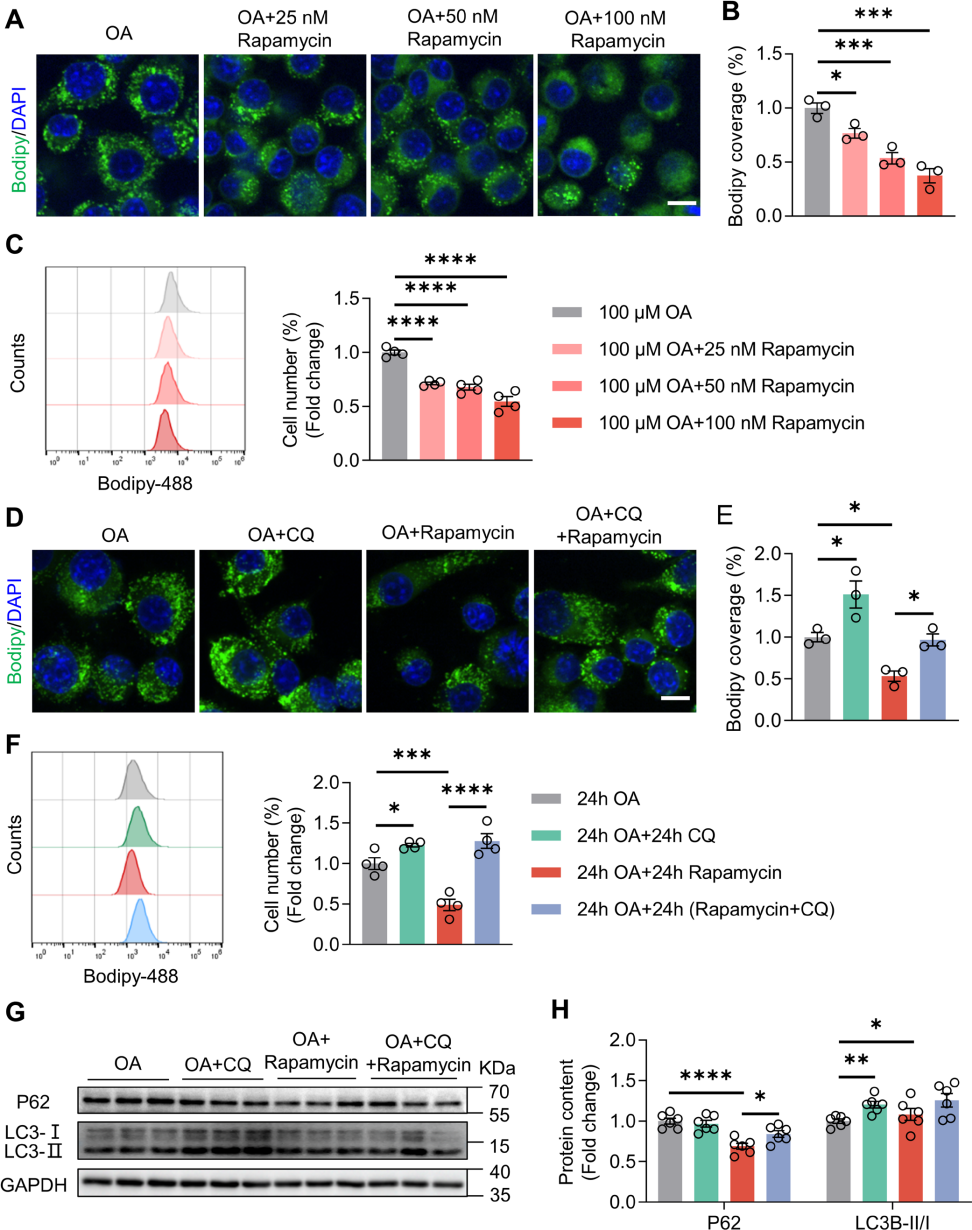
Enhanced lipid droplet clearance by rapamycin treatment via autophagy‐dependent degradation. (A) Confocal images showing BODIPY‐stained OA‐induced lipid droplets in BV2 cells treated with rapamycin (0, 25, 50,100 nM). Scale bar, 10 μm. (B) Quantification of BODIPY fluorescence intensity normalized to OA‐only controls. *n* = 3 independent experiments. OA versus OA + 25 nM Rapamycin: *p* = 0.0463; OA versus OA + 50 nM Rapamycin: *p* = 0.0009; OA versus OA + 100 nM Rapamycin: *p* = 0.0001; one‐way ANOVA. (C) Flow cytometry histograms and quantification of BODIPY fluorescence intensity in BV2 cells co‐treated with OA (100 μM) and rapamycin (0, 25, 50, 100 nM). *n* = 4 independent experiments. OA versus OA + 25 nM Rapamycin: *p* < 0.0001; OA versus OA + 50 nM Rapamycin: *p* < 0.0001; OA versus OA + 100 nM Rapamycin: *p* < 0.0001; one‐way ANOVA. (D) Confocal images showing BODIPY‐stained OA‐induced lipid droplets in BV2 cells treated with OA (100 μM) and rapamycin (100 nM) or chloroquine (CQ, 20 μM). Scale bar, 10 μm. (E) Quantification of BODIPY fluorescence intensity. *n* = 3 independent experiments. OA versus OA + CQ: *p* = 0.0138; OA versus OA + Rapamycin: *p* = 0.0225; OA + Rapamycin versus OA + Rapamycin+CQ: *p* = 0.0477; one‐way ANOVA. (F) Flow cytometry histograms and quantification of BODIPY fluorescence intensity. *n* = 4 independent experiments. OA versus OA + CQ: *p* = 0.0413; OA versus OA + Rapamycin: *p* = 0.0006; OA + Rapamycin versus OA + Rapamycin+CQ: *p* < 0.0001; one‐way ANOVA.(G, H) Immunoblotting and quantification of p62 and LC3 in BV2 cells treated with OA (100 μM) and rapamycin (100 nM) or CQ (20 μM). *n* = 6 independent experiments. P62: OA versus OA versus OA + CQ: *p* = 0.9223; OA versus OA + Rapamycin: *p* < 0.0001; OA + Rapamycin versus OA + Rapamycin+CQ: *p* = 0.0380; LC3B‐II/I: OA versus OA + CQ: *p* = 0.0012; OA versus OA + Rapamycin: *p* = 0.0335; OA + Rapamycin versus OA + Rapamycin+CQ: *p* = 0.2940; one‐way ANOVA. Data are mean ± SEM. *p < 0.05, **p < 0.01, ***p < 0.001, ****p < 0.0001.

Microglial lipid droplet accumulation during aging and AD impairs phagocytic capacity [31, 32]. To determine whether rapamycin enhances Aβ clearance by facilitating lipid droplet degradation, we treated BV2 microglia with increasing doses (50–100 nM). Flow cytometry with BODIPY 493/503 staining revealed a concentration‐dependent reduction in lipid droplet size, with the maximum reduction observed at 100 nM (Figure 6A–C). Mechanistic interrogation using chloroquine (CQ) demonstrated complete blockade of rapamycin's lipid‐clearing effects; CQ increased basal lipid accumulation and abolished the rapamycin‐induced reduction (Figure 6D–F). Western blotting confirmed intact autophagic flux in rapamycin‐treated cells, as evidenced by decreased p62 levels and an increased LC3B‐II/I ratio. Notably, CQ caused p62 sequestration despite an increase in the LC3B‐II/I ratio, whereas rapamycin and CQ co‐treatment prevented p62 degradation (Figure 6G,H). These findings indicate that rapamycin promotes lipid droplet clearance via autophagy‐dependent degradation in the microglia.

### 3.7 Rapamycin Promotes Microglial Recruitment and Autophagic Clearance of Aβ

**FIGURE 7:**
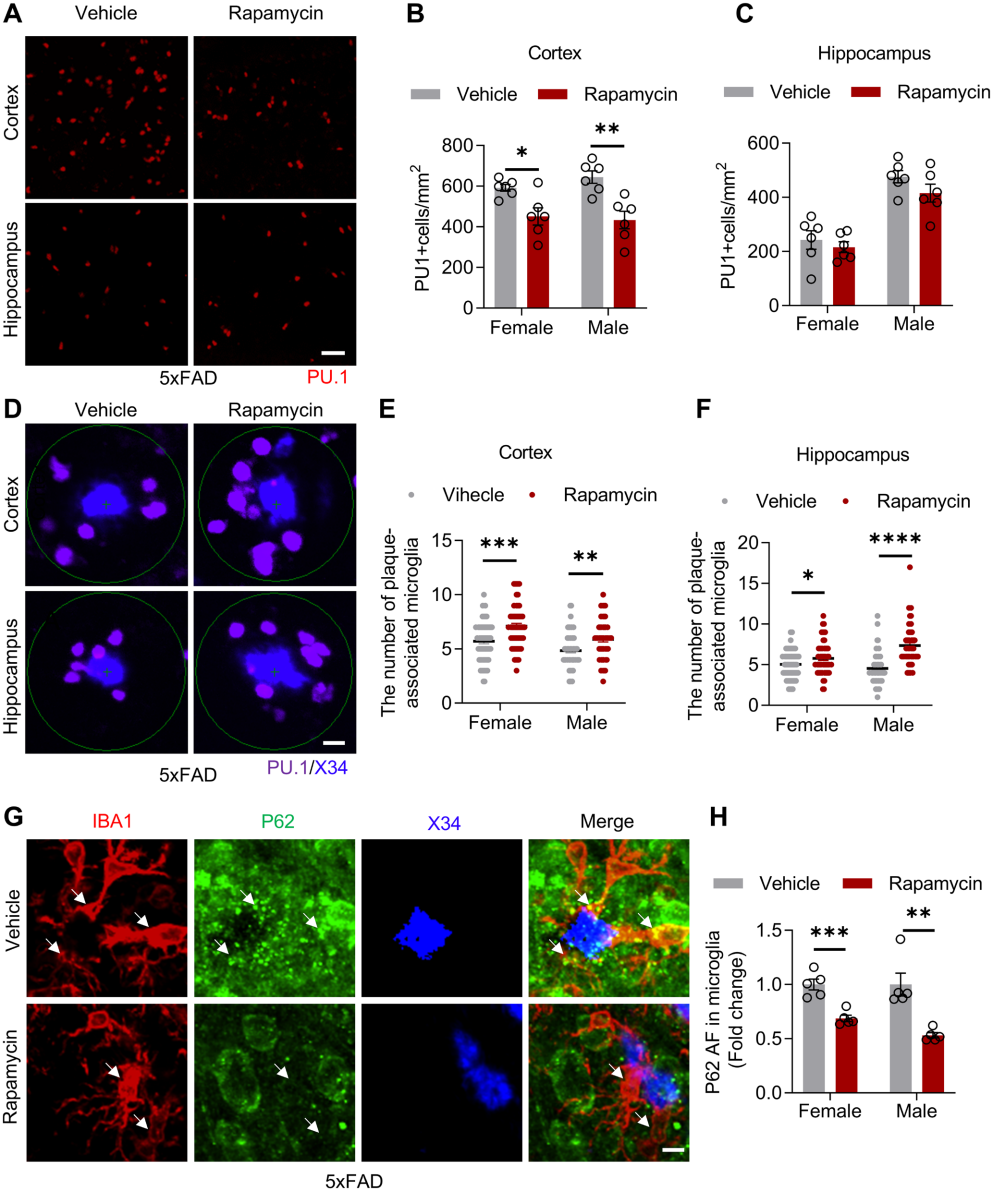
Rapamycin coordinates microglial plaque targeting and autophagy activation to enhance Aβ clearance in 5xFAD mice. (A) Confocal images showing PU.1 (red) staining in the cortex and hippocampus of vehicle‐ or rapamycin‐treated female and male 5xFAD mice. Scale bar, 50 μm. (B, C) Density of PU.1^+^ cells in the cortex (B) and hippocampus (C) of the mice. *n* = 6 mice per group. Cortex female: *T*_(10)_ = 3.081, *p* = 0.0116; cortex male: *T*_(10)_ = 4.030, *p* = 0.0024; hippocampus female: *T*_(10)_ = 0.6767, *p* = 0.5140; hippocampus male: *T*_(10)_ = 1.512, *p* = 0.1615, unpaired *t*‐test. (D) Confocal images showing plaque (X34, blue)‐associated microglia (PU.1, purple). Scale bar,10 μm. (E, F) Number of plaque‐associated microglia in the cortex (E) and hippocampus (F). *n* = 6 mice per group. Ten plaques per mouse were selected for the statistical analysis. Cortex female: *T*_(118)_ = 3.962, *p* = 0.0001; cortex male: *T*_(118)_ = 3.306, *p* = 0.0013; hippocampus female: *T*_(10)_ = 2.143, *p* = 0.0342; unpaired *t*‐test. Hippocampus male: *P* < 0.0001, Mann–Whitney test. (G) Confocal images and (H) quantification showing p62 (red) in plaque (X34, blue)‐associated microglia (Iba1, green) in the cortex. Scale bar,10 μm. Female: *T*_(8)_ = 5.324, *p* = 0.0007; unpaired *t*‐test. Male: *p* = 0.0079, Mann–Whitney test. *n* = 5 mice per group. Data are mean ± SEM. *p < 0.05, **p < 0.01, ***p < 0.001, ****p < 0.0001.

Based on evidence that rapamycin clears lipid droplets via autophagic flux in vitro, we examined its effects on microglial function in AD models. PU.1 immunohistochemistry revealed significantly reduced cortical microglial cell density in rapamycin‐treated 5xFAD and female hAPP^NL‐G‐F^ mice, whereas hippocampal densities remained unaffected (Figure 7A–C; Figure S7A–C). Notably, X34/PU.1 co‐staining demonstrated enhanced microglial clustering around cortical/hippocampal plaques in treated mice (Figure 7D–F; Figure S7D–F), concomitant with markedly reduced p62 accumulation in peri‐plaque microglia (Figure 7G,H; Figure S7G,H).

**FIGURE 8:**
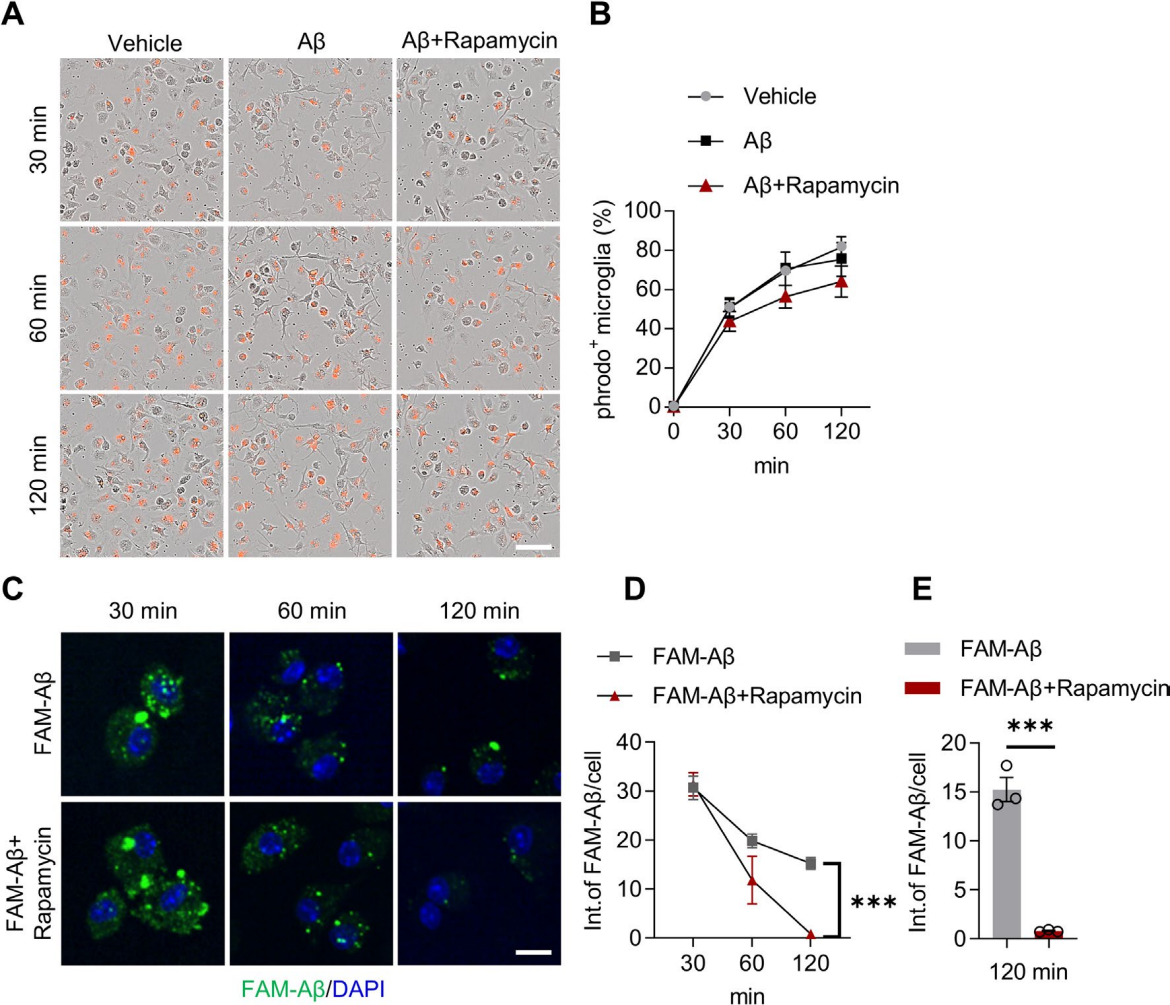
Rapamycin enhances Aβ degradation in primary microglia. (A) Images showing BV2 cells treated with pHrodo Red Zymosan BioParticles (orange), rapamycin, and Aβ_1‐42_ at 30, 60, and 120‐min. Scale bar, 50 μm. (B) Quantification of the percentage of pHrodo‐positive BV2 cells. *n* = 3 independent experiments. 30 min: Vehicle versus Aβ: *p* > 0.9999, Aβ versus Aβ + Rapamycin: *p* = 0.5787; 60 min: Vehicle versus Aβ: *p* = 0.9836, Aβ versus Aβ + Rapamycin: *p* = 0.2625; 120 min: Vehicle versus Aβ: *p* = 0.8190, Aβ versus Aβ + Rapamycin: *p* = 0.5454; one‐way ANOVA. (C) Confocal images of BV2 cells with or without rapamycin treatment at 30, 60, and 120 min after FAM‐Aβ removal (green). Scale bar, 10 μm. (D) Quantification of FAM‐Aβ fluorescence intensity in BV2 cells at different time points after removal. *n* = 3 independent experiments. 30 min: *T*_(4)_ = 0.2052, *p* = 0.8474, unpaired *t*‐test; 60 min: *p* = 0.1000, Mann–Whitney test; 120 min: *T*_(4)_ = 11.66, *p* = 0.0003; unpaired *t*‐test. (E) Comparison of FAM‐Aβ fluorescence intensity in BV2 cells at 120 min after removal. *n* = 3 independent experiments. *t*_(4)_ = 11.66, *p* = 0.0003; unpaired *t*‐test. Data are mean ± SEM. ***p < 0.001.

To dissect the phagocytic versus degradative mechanisms, primary microglia were treated with pHrodo Red Zymosan BioParticles or FAM‐labeled Aβ fibrils. Rapamycin pretreatment (24 h) did not alter pHrodo‐Aβ uptake at 30–120 min (Figure 8A,B) but significantly accelerated FAM‐Aβ degradation at 120 min (Figure 8C–E). These coordinated changes indicate that rapamycin recruits microglia to Aβ plaques and enhances Aβ degradation via autophagic pathways without directly stimulating phagocytic uptake, thereby improving Aβ clearance.

## 4 Discussion

AD is characterized by a critical imbalance between Aβ production and clearance, with clearance being the primary driver of sporadic AD pathology [2, 33]. Our findings demonstrate that early rapamycin administration (at 2 months) effectively reduced plaque burden and stabilized behavioral performance in two complementary AD models. These results align with the “window of opportunity” hypothesis, suggesting that mTOR inhibition is most effective when initiated during the seeding phase of amyloidosis [12]. Rapamycin appears to prevent the transition from soluble Aβ to insoluble aggregates, reinforcing the therapeutic potential of enhancing clearance pathways before irreversible neurodegeneration occurs [34, 35].

Rapamycin is a well‐known inducer of autophagy, a lysosomal process essential for Aβ homeostasis [36, 37, 38], and its role in AD has been debated previously. Some reports have suggested that excessive autophagic stress may paradoxically exacerbate plaque formation [39, 40]. However, our study provides clarity by identifying microglial functional rejuvenation as the primary mediator of rapamycin benefits. We observed that rapamycin not only increased general autophagic markers but also specifically enhanced microglial recruitment to plaques and promoted a degradative phenotype. The observed reduction in p62 levels within plaque‐associated microglia indicates an acceleration of autophagic flux, effectively bypassing the “stalled” autophagy often observed in AD models. This restoration of degradative proteostasis was further validated by our in vitro data, which showed that rapamycin directly boosted the microglial Aβ‐degrading capacity.

A key highlight of our mechanistic investigation was the intersection of lipid metabolism and microglial functions. Transcriptomic enrichment analysis indicated robust modulation of lipid pathways, which are essential for Aβ clearance [41]. Consistent with its role in enhancing fatty acid oxidation and suppressing lipid droplet accumulation in the periphery [42, 43, 44], rapamycin treatment in our study reduced lipid droplet accumulation in microglia, a pathological state known to impair phagocytic efficiency [45]. By inducing autophagy‐mediated lipid degradation, rapamycin “clears the brakes” on microglial motility and engulfment. This cohesive model, in which lipid homeostasis supports autophagic flux, explains how rapamycin restores the CNS immune environment in patients with MS.

Perhaps the most significant insight of this study is the sex‐specific efficacy of rapamycin in the 5xFAD mice. The pronounced histological and behavioral improvements in female mirror findings in APOE4‐targeted studies [46] may stem from an interaction between the aggressive baseline pathology of female 5xFAD mice [47] and the sexually dimorphic pharmacokinetics of rapamycin [28, 48, 49]. Interestingly, the subtle response in the hAPP^NL‐G‐F^ model suggests that the differential effects of rapamycin are amplified in high‐pathology contexts, such as AD. This underscores that while mTOR‐mediated lifespan extension may be universal [50], its therapeutic efficacy in neurodegeneration is deeply modulated by sex‐specific disease biology.

Our positive results contrast with a few reports, such as the increased Aβ levels observed in female Tg2576 mice after short‐term treatment [51]. These discrepancies are likely attributable to differences in mouse models (PDAPP vs. Tg2576 vs. 5xFAD), treatment duration, and specific timing of the intervention [13, 52, 53, 54, 55, 56]. Our study suggests that sustained early phase treatment is superior to late‐stage “rescue” attempts. However, several questions remain. Our study exclusively focused on Aβ pathology and used a single drug dose. Future investigations should explore the impact of rapamycin on tau pathology, synaptic pruning, and dose–response curves across various disease stages to fully define its clinical utility in AD.

## 5 Conclusions

In summary, early rapamycin treatment restores microglial function by integrating lipid homeostasis and autophagic clearance. This study provides the first integrated in vivo and in vitro evidence that rapamycin rejuvenates microglial degradation, particularly in a sex‐dependent manner. Our findings highlight the need to incorporate sex as a biological variable when developing mTOR‐targeted AD therapies.

## Author Contributions

**Shihui Guo:** resources, investigation, validation, methodology, visualization, writing – original draft, writing – review and editing. **Weishan Fu:** investigation, resources, methodology, validation. **Yating Wang:** methodology, validation, and visualization. **Qi Liu:** investigation. Jiaxin Li: methodology. **Kai Guo:** methodology, data curation, software. **Hongsheng Zhang:** conceptualization, resources, validation, supervision, funding acquisition, writing – review and editing.

## Funding

This work was supported by the STI2030‐Major Projects‐2021ZD0202400, National Natural Science Foundation of China grants 82271472 (H.S.Z), Lingang Laboratory (Grant No. LG‐GG‐202401‐ADAD060100, and LG‐GG‐202401‐ADA060200), Science and Technology Research Program of Chongqing Municipal Education Commission (KJQN202200479, KJQN202500456), Natural Science Foundation of Chongqing (CSTB2022NSCQ‐LZX0033), CQMU Program for Youth Innovation in Future Medicine (W0158), Chongqing Graduate Research Innovation Project (Grant No. CYS23377), and Natural Science Foundation of Fujian Province (Grant No. 2021J01016).

## Ethics Statement

All animal procedures employed in this study were conducted in accordance with the National Institutes of Health Guidelines for the Care and Use of Laboratory Animals and were approved by the Institutional Animal Care and Use Committee of Chongqing Medical University (IACUC‐CQMU‐2024‐0931). All the cell lines used in this study were obtained from commercial sources.

## Conflicts of Interest

All the authors have reviewed and approved the manuscript.

## Supporting information


**Figures S1–S7:** cns70807‐sup‐0001‐FiguresS1‐S7.pdf.

## Data Availability

The bulk RNA‐seq data generated in this study were deposited in the GEO database under the accession number GSE304662. All other supporting datasets are available from the corresponding author upon reasonable request.
