# Supplementary material for: Rapamycin Reduces Amyloid‐β Plaques and Improves Behavioral Performance in a Sex‐Dependent Manner in Mouse Models of Amyloidosis
Source: CNS Neurosci Ther. 2026 Feb 26;32(3):e70807. doi: 10.1002/cns.70807 (PMC12945924; doi:10.1002/cns.70807)
Supplement: Supplementary file 1 — Figures S1–S7: cns70807‐sup‐0001‐FiguresS1‐S7.pdf. [file CNS-32-e70807-s001.pdf]

Figure S1

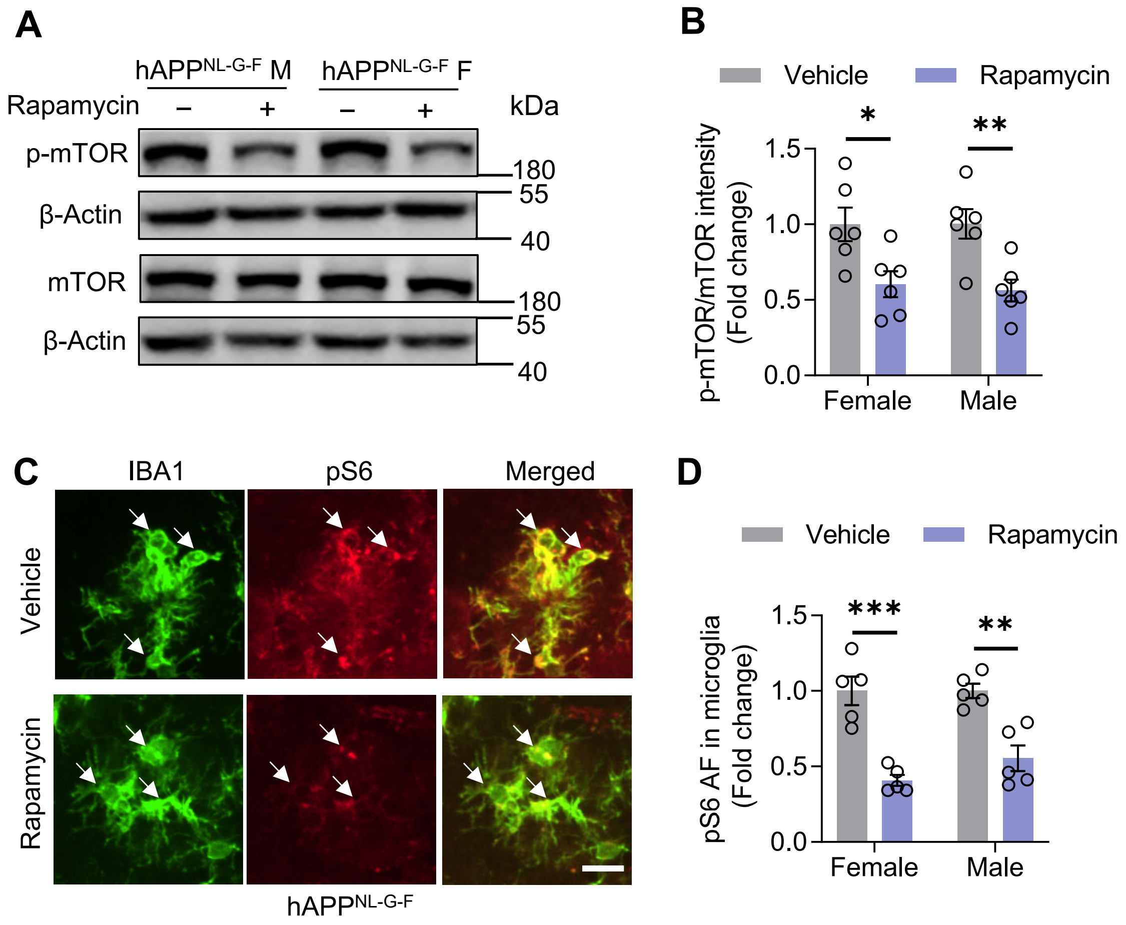

Figure S1. Reduced mTOR activity and downstream signaling in rapamycin treated hAPP$^{NL\text{-}G\text{-}F}$mice.

(A, B) Immunoblot analysis and quantification of total mTOR and p-mTOR in the cortex of vehicle-or rapamycin-treated hAPP$^{NL\text{-}G\text{-}F}$mice. n = 6 mice per group. Female: $t_{(10)}$ =2.829, *P*=0.0179; male: $t_{(10)}$ =3.627, *P*=0.0046, unpaired t test. **(C, D)**Confocal images and quantification of pS6 (red) in Iba1-positive microglia (green) located near Aβ plaque vehicle-or rapamycin-treated hAPP$^{NL\text{-}G\text{-}F}$ mouse brains. Scale bar,10 μm. n = 5 mice per group. Female: $t_{(8)}$ =5.860*,* *P*=0.0004; male: $t_{(8)}$ =4.558, *P*=0.0019, unpaired t test. Data are mean ±SEM. **P* < 0.05, ***P* < 0.01, ****P* < 0.001.

Figure S2

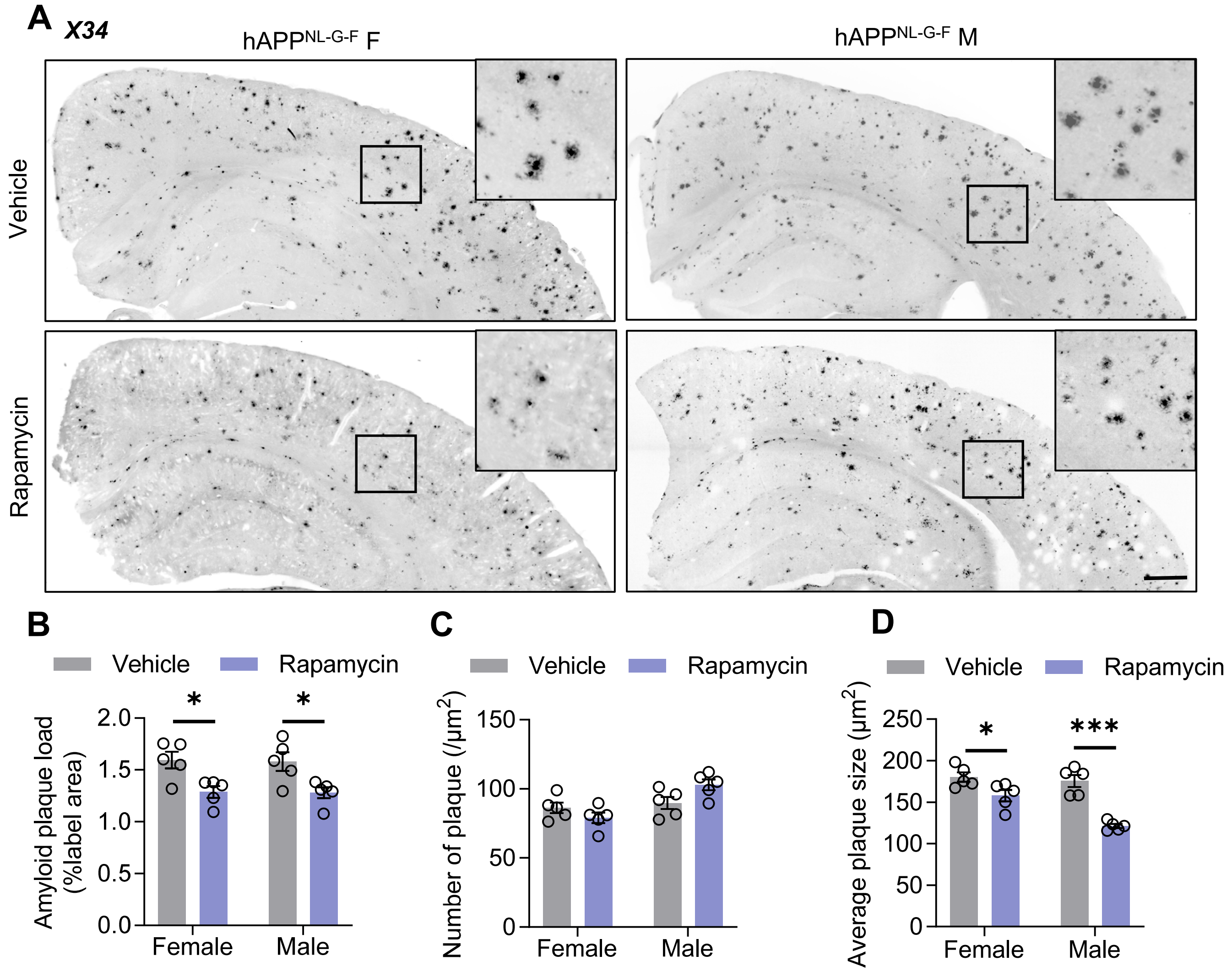

Figure S2. Ameliorated Aβ pathology in rapamycin treated hAPP$^{NL\text{-}G\text{-}F}$ mice.

(A) Confocal images showing Aβ plaques (X34 staining) in the brains of vehicle-or rapamycin-treated female and male hAPP$^{NL\text{-}G\text{-}F}$ mice. Scale bar, 200 μm. **(B-D)** Quantification of Aβ plaque load **(B)**, density **(C)**, and average size **(D)**in the brains of vehicle-or rapamycin-treated female and male hAPP$^{NL\text{-}G\text{-}F}$ mice. n = 5 mice per group. Amyloid plaque load, female: $t_{(8)}$ =3.161, *P*=0.0134, male: $t_{(8)}$ =2.863, *P*=0.0210; number of plaque, female: $t_{(8)}$ =1.318, *P*=0.2239, male: $t_{(8)}$ =2.216, *P*=0.0575; average plaque size, female: $t_{(8)}$ =2.492, *P*=0.0374, male: $t_{(8)}$ =7.038, *P*=0.0001; unpaired t test. Data are mean ±SEM. **P* < 0.05, . ****P* < 0.001.

Figure S3

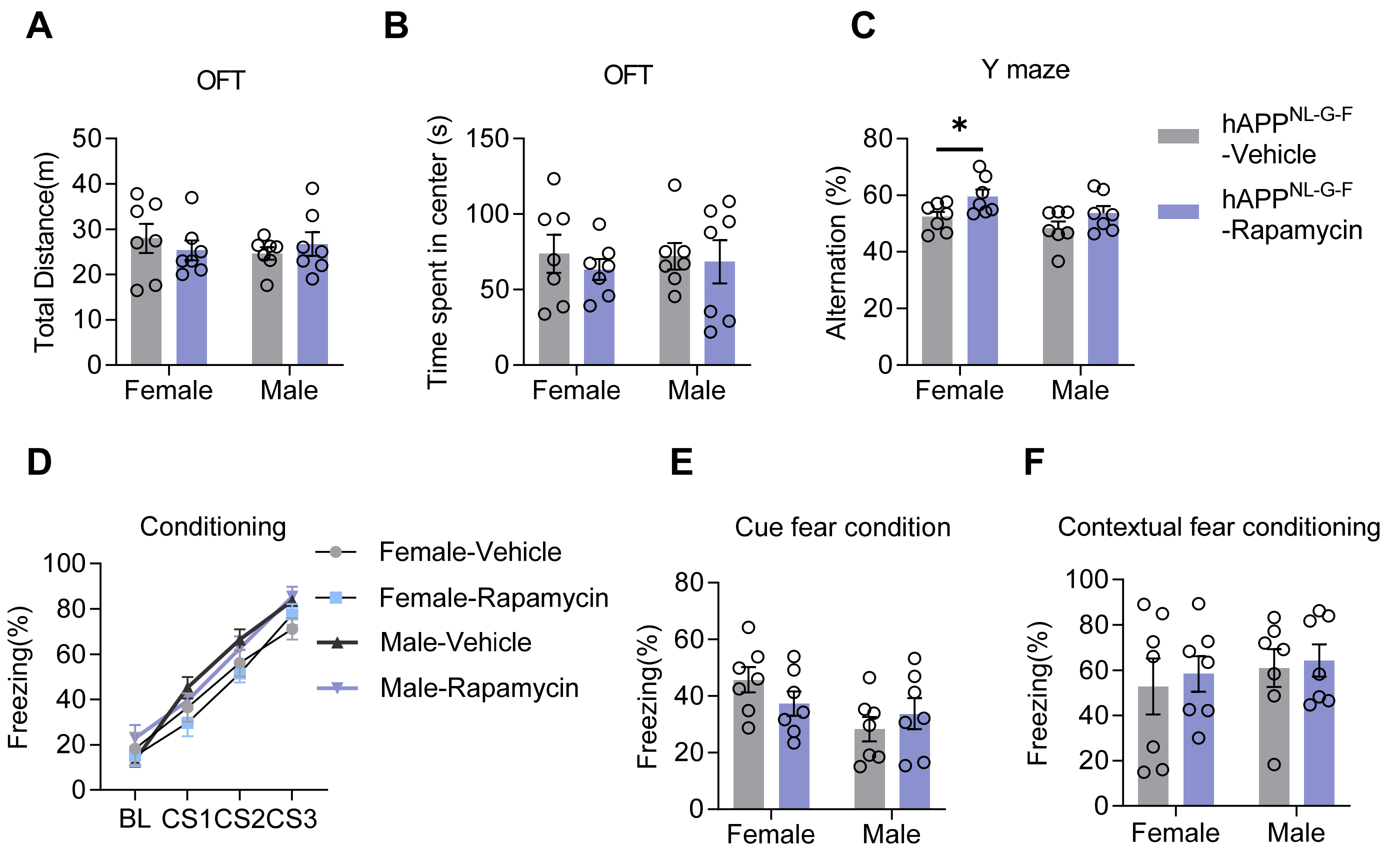

Figure S3. Mitigated cognitive impairment in rapamycin treated hAPP$^{NL\text{-}G\text{-}F}$ mice.

(A, B) Total distance traveled **(A)** and time spent in center zone **(B)**during open field test for vehicle-or rapamycin-treated hAPP$^{NL\text{-}G\text{-}F}$ mice. Total distance Female: $t_{(12)}$ =0.6963, *P*=0.4995; Total distance Male: $t_{(12)}$ =0.7024, *P*=0.4958; Time spent in center Female : $t_{(12)}$ =0.7307, *P*=0.4790; Time spent in center Male : $t_{(12)}$ =0.2135, *P*=0.8345; unpaired t test. **(C)**Percentage of spontaneous alternations during Y-maze test for vehicle-or rapamycin-treated hAPP$^{NL\text{-}G\text{-}F}$mice. Female: $t_{(12)}$ =2.349, *P*=0. 0368; unpaired t test; Male: $t_{(12)}$ =1.5245, *P*=0.1535; unpaired t test. **(D-F)** Percentage of freezing during fear conditioning training **(D)**, cued fear conditioning **(E)**, contextual fear conditioning **(F)**in vehicle-or rapamycin-treated hAPP$^{NL\text{-}G\text{-}F}$ mice. Cued fear conditioning Female : $t_{(12)}$ =1.359, *P*= 0.1990; Cued fear conditioning Male : $t_{(12)}$ =0.7862, *P*=0.4470; contextual fear conditioning Female : $t_{(12)}$ =0.3793, *P*= 0.7111; contextual fear conditioning Male : $t_{(12)}$ =0.2959, *P*= 0.7724; n = 7 mice per group. Data are mean ±SEM. **P* < 0.05.

Figure S4

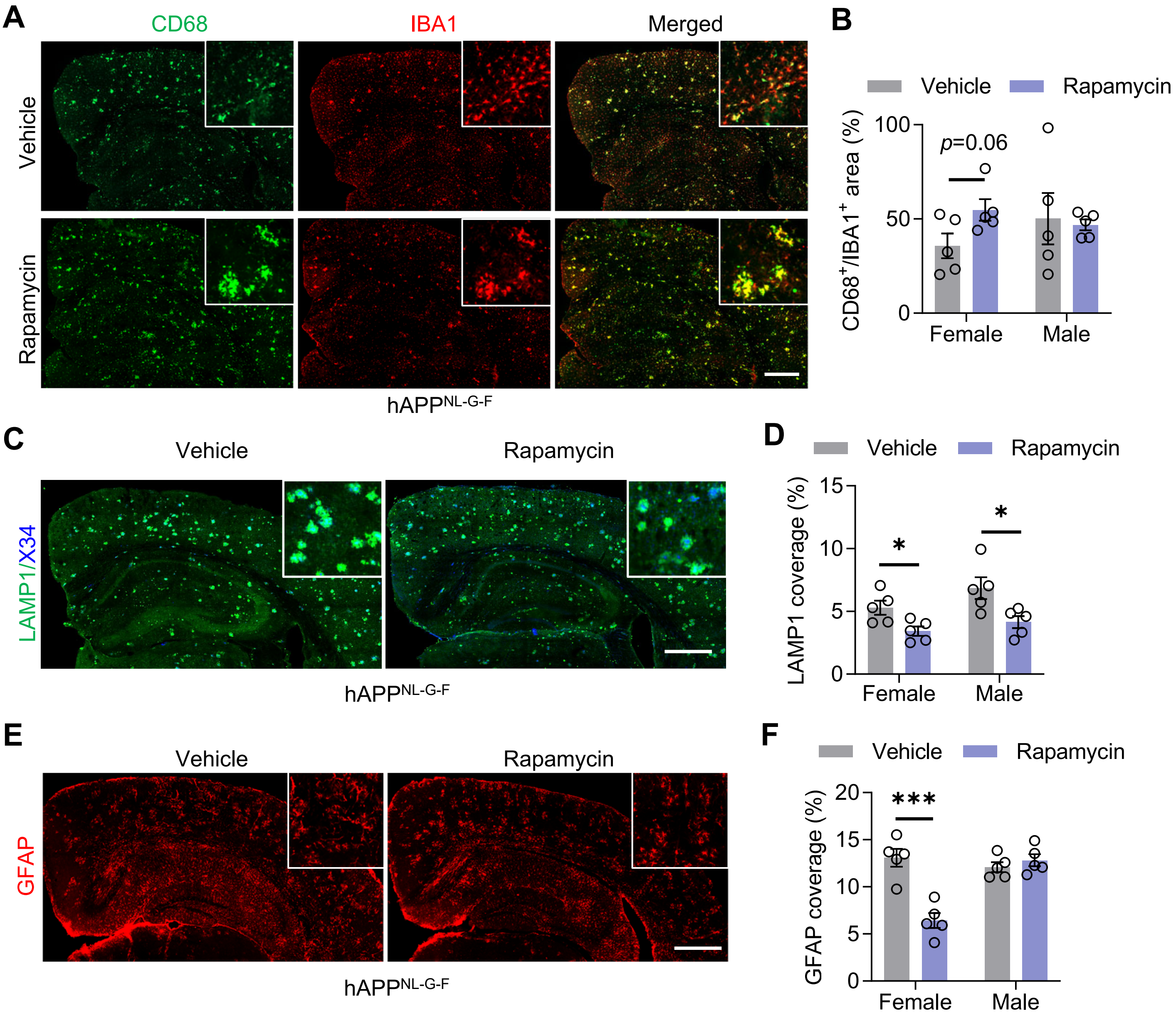

Figure S4. Reduced amyloid plaque-associated toxicity in rapamycin treated hAPP$^{NL\text{-}G\text{-}F}$ mice.

(A) Confocal images showing CD68 (green) and Iba1 (red) staining in the brains of vehicle-or rapamycin-treated female and male hAPP$^{NL\text{-}G\text{-}F}$ mice. Scale bar, 500 μm. **(B)**Quantification of CD68+/Iba1+area ratio in vehicle-or rapamycin-treated female and male hAPP$^{NL\text{-}G\text{-}F}$ mice. n = 5 mice per group. Female: $t_{(8)}$ =2.177, *P*=0.0611; male: $t_{(8)}$ =0.2351, *P*=0.8200, unpaired t test. **(C)**Confocal images showing LAMP1 (green) staining. Scale bar, 500 μm. **(D)**Quantification of the LAMP1+area in vehicle-or rapamycin-treated female and male hAPP$^{NL\text{-}G\text{-}F}$ mice. n = 5 mice per group. Female: $t_{(8)}$ =2.776, *P*=0.0241; male: $t_{(8)}$ =2.735, *P*=0.0256, unpaired t test. **(E)**Confocal images showing GFAP (red) staining. Scale bar, 500 μm. **(F)**Quantification of the GFAP+area vehicle-or rapamycin-treated female and male hAPP$^{NL\text{-}G\text{-}F}$ mice. n = 5 mice per group. Female: $t_{(8)}$ =5.437, *P*=0.0006; male: $t_{(8)}$ =0.8683, *P*=0.4105, unpaired t test. Data are mean ±SEM. * $P < 0.05$, *** $P < 0.001$.

Figure S5

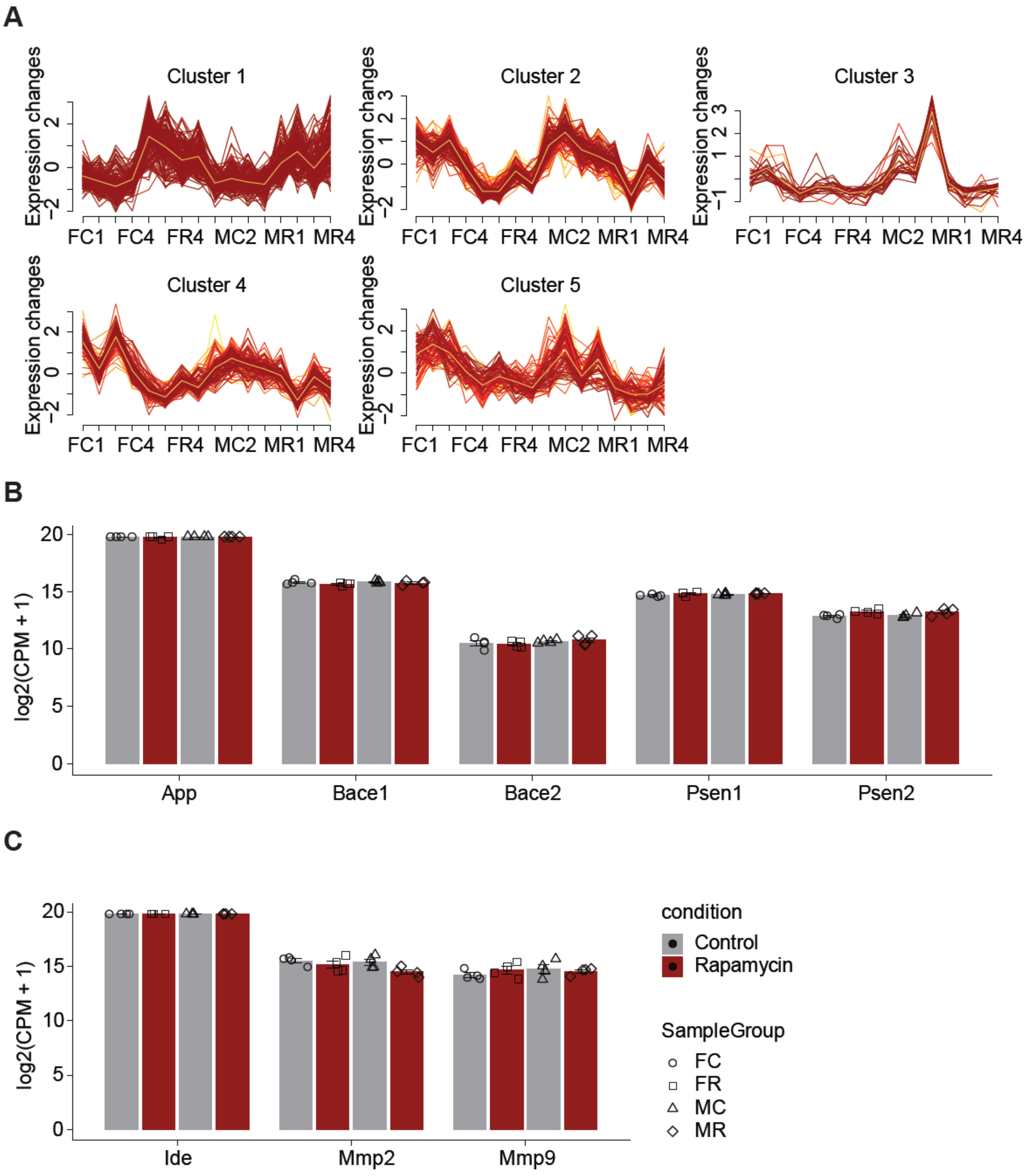

Figure S5. Clustering of DEGs using Mfuzz(v2.66.0).

(A)DEGs were grouped into five distinct co-expression modules (Cluster 1-5) based on their expression patterns across different groups. n = 4 mice per group. Filtering criteria: |log2 FC| > 0.5 and p.adj<0.05. **(B)** mRNA levels of several key genes involved in Aβ metabolism in the brains of vehicle-or rapamycin-treated female and 5xFAD mice measured by RNA-seq. **(C)** mRNA levels of several Aβ-degrading proteases in the brains of vehicle-or rapamycin-treated female and 5xFAD mice measured by RNA-seq. FC, female vehicle-treated 5xFAD; FR, female rapamycin-treated 5xFAD; MC, male vehicle-treated 5xFAD; MR, male rapamycin-treated 5xFAD.

Figure S6

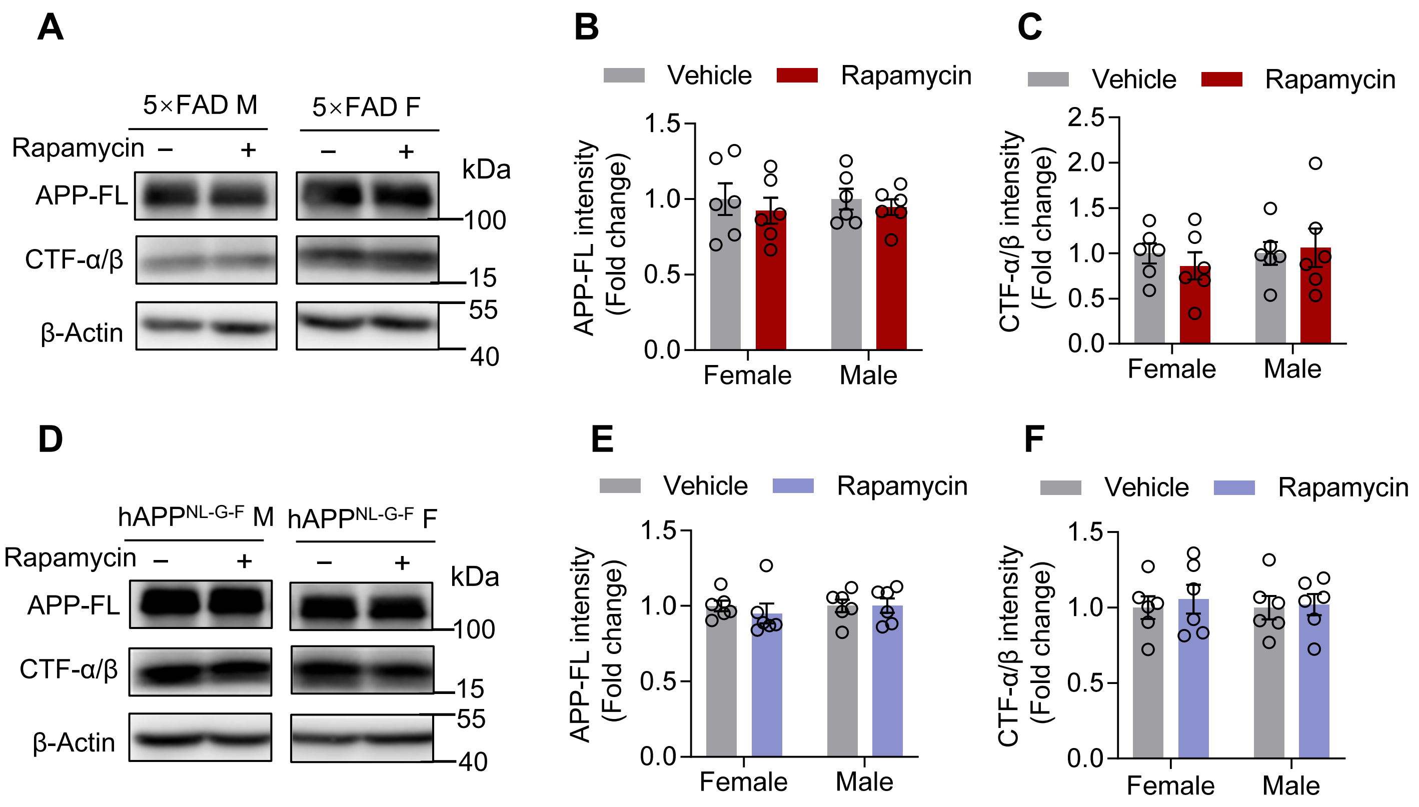

Figure S6. Rapamycin does not effect Aβ production.

(A-C) Immunoblot analysis and quantification of APP and CTF-α/β levels in the cortex of vehicle-or rapamycin-treated 5xFAD mice. n = 6 mice per group. APP-FL female: $t_{(10)}$ =0.5688, *P*=0.5821; male: $t_{(10)}$ =0.6162, *P*=0.5515, unpaired t test. CTF-α/β female: : $t_{(10)}$ =0.7442, *P*=0.4739; male: $t_{(10)}$ =0.2478, *P*=0.8093, unpaired t test. **(D-F)** Immunoblot analysis and quantification of APP and CTF-α/β levels in the cortex of vehicle-or rapamycin-treated hAPP$^{NL\text{-}G\text{-}F}$mice. n = 6 mice per group. APP-FL female: $t_{(10)}$ =0.6810, *P*= 0.5113; male: $t_{(10)}$ =0.03536, *P*=0.9725, unpaired t test. CTF-α/β female: $t_{(10)}$ =0.4616, *P*=0.6543; male: $t_{(10)}$ =0.1942, *P*=0.8499, unpaired t test. Data are mean ±SEM.

Figure S7

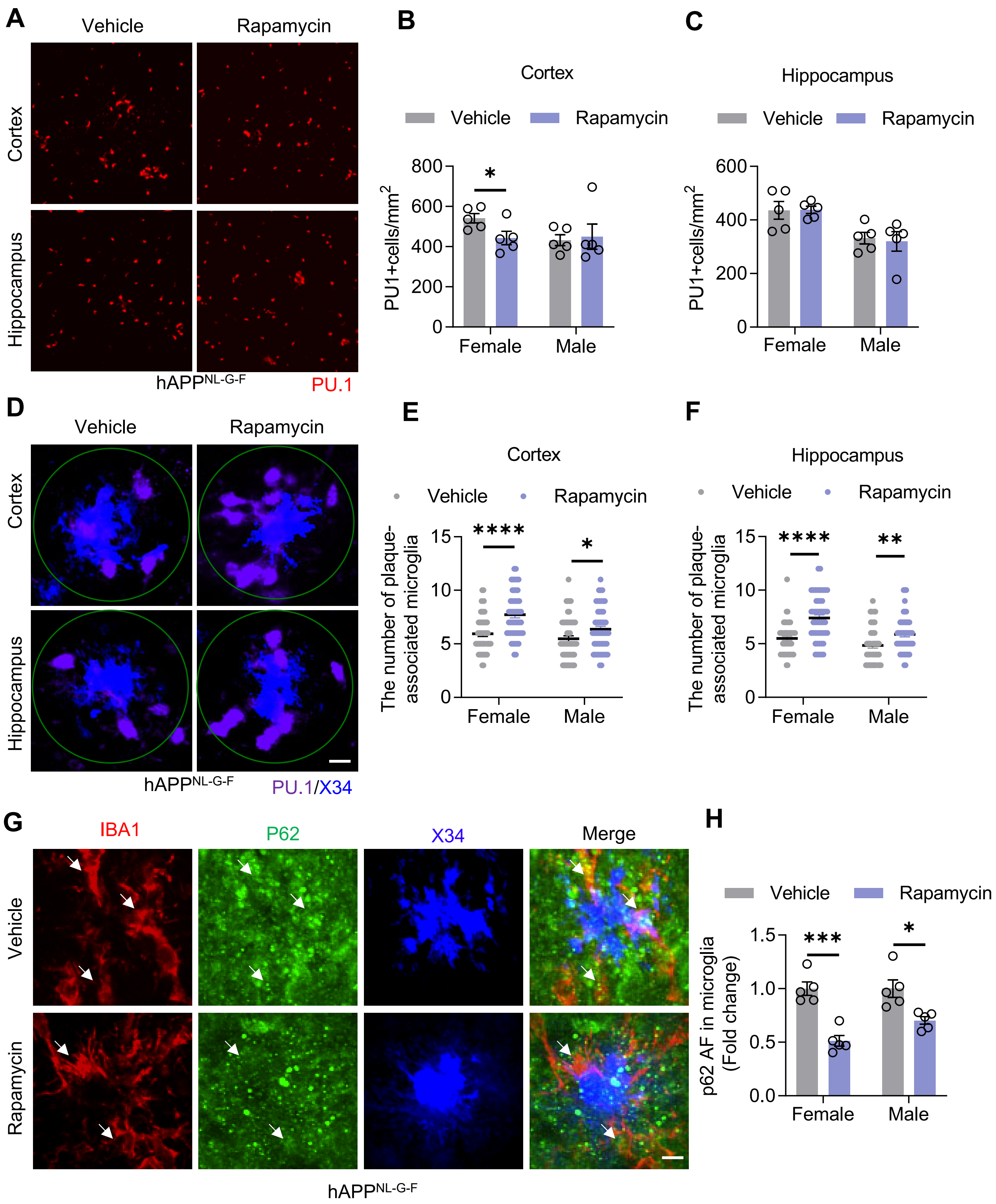
A
Vehicle
Rapamycin
Cortex
Hippocampus
hAPP NL-G-F
PU.1
B
Cortex
Vehicle
Rapamycin
PU1+cells/mm2
800
600
400
200
0
*
Female
Male
C
Hippocampus
Vehicle
Rapamycin
PU1+cells/mm2
600
400
200
0
Female
Male
D
Vehicle
Rapamycin
Cortex
Hippocampus
hAPP NL-G-F
PU.1/X34
E
Cortex
Vehicle
Rapamycin
The number of plaque-associated microglia
15
10
5
0
****
*
Female
Male
F
Hippocampus
Vehicle
Rapamycin
The number of plaque-associated microglia
15
10
5
0
****
**
Female
Male
G
IBA1
P62
X34
Merge
Vehicle
Rapamycin
hAPP NL-G-F
H
Vehicle
Rapamycin
p62 AF in microglia (Fold change)
1.5
1.0
0.5
0.0
***
*
Female
Male

Figure S7. Rapamycin coordinates microglial plaque targeting and autophagy activation to enhance Aβ clearance in hAPP$^{NL\text{-}G\text{-}F}$ mice.

(A) Confocal images showing PU.1 (red) staining in the cortex and hippocampus of vehicle-or rapamycin-treated female and male hAPP$^{NL\text{-}G\text{-}F}$mice. Scale bar, 50 μm. **(B, C)** Quantification of PU.1+ cell density in the cortex **(B)**and hippocampus **(C)**of vehicle-or rapamycin-treated female and male hAPP$^{NL\text{-}G\text{-}F}$mice. n = 5 mice per group. Cortex female: $t_{(8)}$ =2.428, *P*=0.0413, unpaired t test ; cortex male: *P*=0.833, Mann Whitney test; hippocampus female: $t_{(8)}$ =0.05009, *P*=0.9613, unpaired t test ; hippocampus male: *P*=0.8810, Mann Whitney test. **(D)** Confocal images showing plaque (X34, blue)-associated microglia (PU.1, purple) in the cortex and hippocampus of vehicle-or rapamycin-treated female and male hAPP$^{NL\text{-}G\text{-}F}$mice. Scale bar,10 μm. **(E, F)** Quantificationofplaque-associated microglia in the cortex **(E)** and hippocampus **(F)**of vehicle-or rapamycin-treated female and male 5xFAD mice. n = 5 mice per group. 10 plaques per mouse were selected for statistical analysis. Cortex female: $t_{(98)}$ =4.600, $P < 0.0001$; cortex male: $t_{(98)}$ =2.178, *P*=0.0318; hippocampus female: $P < 0.0001$, Mann Whitney test; hippocampus male: $t_{(98)}$ =3.044, *P*=0.0030, unpaired t test. **(G)**Confocal images showing p62 (red) in plaque (X34, blue)-associated microglia (Iba1, green) in the cortex of vehicle-or rapamycin-treated female and male hAPP$^{NL\text{-}G\text{-}F}$mice. Scale bar, 10 μm. **(H)** Quantification of p62 (red) area in-associated microglia in the cortex of vehicle-or rapamycin-treated female and male hAPP$^{NL\text{-}G\text{-}F}$mice. Scale bar,10 μm. n = 5 mice per group. Female: $t_{(8)}$ =6.126, *P*=0.0003; male: $t_{(8)}$ =3.305, *P*=0.0108, unpaired t test. Data are mean ±SEM. *$P < 0.05$, **$P < 0.01$, ***$P < 0.001$, ****$P < 0.0001$.
